# Diversity of dietary protein patterns across Europe – Impact on nutritional quality and environmental sustainability

**DOI:** 10.1016/j.crfs.2025.101019

**Published:** 2025-03-04

**Authors:** Merel C. Daas, Pieter van 't Veer, Elisabeth H.M. Temme, Anneleen Kuijsten, Mirjana Gurinović, Sander Biesbroek

**Affiliations:** a Division of Human Nutrition and Health, Wageningen University & Research, 6700 AA, Wageningen, the Netherlands; b Centre for Prevention, Lifestyle and Health, Department for Healthy and Sustainable Nutrition, National Institute for Public Health and the Environment (RIVM), 3721 MA, Bilthoven, the Netherlands; c Centre of Research Excellence in Nutrition and Metabolism, Institute for Medical Research, National Institute of Republic of Serbia, University of Belgrade, 11000, Belgrade, Serbia; d Capacity Development in Nutrition (CAPNUTRA), Belgrade, Serbia

**Keywords:** Protein transition, Protein sources, Protein consumption profile, Nutrient adequacy, Environmental footprint, Sustainable diet

## Abstract

Transitioning from animal-based to plant-rich diets could potentially improve both human and planetary health, but a thorough understanding of the protein component in the diet is essential. This research aimed to identify dietary protein patterns in the European adult population and evaluate differences in nutritional quality and environmental sustainability. Individual-level food consumption data were obtained from 25 European countries (40,101 participants, 18–64 years), available from the EFSA Comprehensive European Food Consumption Database. We applied statistical clustering to classify individuals according to their consumption of 24 protein source food groups. The patterns were evaluated for nutrient requirements, the Nutrient Rich Diet (NRD) 15.3 score, greenhouse gas emissions (GHGE) and land use (LU). Six patterns emerged: *Common* (42.2%), *Fast-food* (19.5%), *Traditional* (14.8%), *Health-conscious* (12.0%), *Milk-rich* (9.8%) and *Plant-forward* (1.6%), with country-specific variations. Most patterns obtained 64–69% of their protein intake from animal products, except for the *Plant-forward* pattern (52%). The *Plant-forward* pattern achieved the highest NRD15.3 (+11%), and lowest GHGE (−20%) and LU (−25%) compared to the population average and was most commonly consumed in Austria, Finland, Spain, Portugal and Belgium (4.1–4.5%). The *Health-conscious* pattern also scored high in nutritional quality (NRD15.3: +9%), whereas the *Traditional* pattern showed higher environmental impacts (GHGE: +5%, LU: +7%). These findings highlight the diversity of dietary protein patterns across Europe, each with unique nutritional profiles and varying environmental impacts. The *Plant-forward* pattern provides a promising example for healthier, more sustainable diets, but tailored approaches that consider the cultural and demographic contexts of individual countries are needed to optimize health and environmental outcomes for all patterns.

## Highlights

• Six unique dietary protein patterns were identified across 25 European countries.
• 42.2% of Europeans followed a *Common* pattern and only 1.6% a *Plant-forward* pattern.
• 52% of protein was animal-based in the *Plant-forward* pattern vs ∼67% in the others.
• *Plant-forward* pattern had highest nutrient quality and lowest environmental impact.
• Tailored strategies are needed to shift European diets towards more plant foods.

## Graphical abstract

**Figure:**
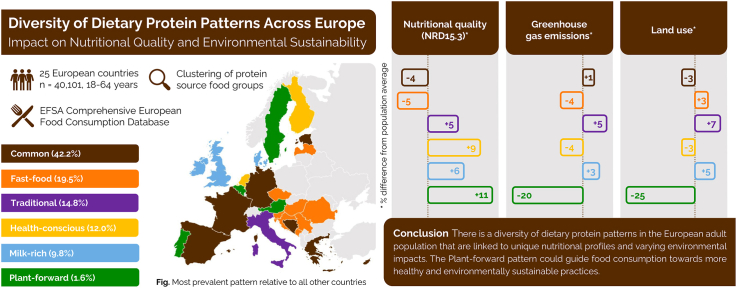

## 1 Introduction

A transition from diets primarily based on animal protein towards diets rich in plant and/or alternative protein has gained increasing interest as a solution to reduce environmental impacts and improve human health (Aiking and de Boer, 2020; Fasolin et al., 2019; Lonnie and Johnstone, 2020; Henchion et al., 2017). Production and consumption of proteins are major determinants of the environmental impacts of our diets, with animal-based protein foods generally inducing higher impacts per kilogram than plant-based protein foods (Clark et al., 2019; Aiking, 2014). Additionally, consumption of animal-based protein foods, such as red and processed meat, have been associated with increased risks of non-communicable diseases and premature mortality (Farvid et al., 2021; Abete et al., 2014; Wang et al., 2016).

Several European countries, such as Belgium, the Netherlands and Denmark, have proposed a rebalancing of animal and plant protein in the diet as a step towards more sustainable food systems (Vlaamse overheid, 2021; Raad voor de leefomgeving en infrastructuur, 2018; Ministry of Food and Agriculture and Fisheries of Denmark, 2023). In high-income countries, approximately 60–70% of dietary protein currently originates from animal sources (Pasiakos et al., 2015; EFSA Panel on Dietetic Products and Nutrition and Allergies (NDA), 2012; Halkjær et al., 2009). On average, meat and dairy products provide most protein in the diet, followed by grains, fish and seafood, eggs, and other plant foods (Pasiakos et al., 2015; EFSA Panel on Dietetic Products and Nutrition and Allergies (NDA), 2012; Halkjær et al., 2009). However, large variations in the production and consumption of protein sources exist between countries because of differences in food cultures, agricultural practices and economic conditions throughout history (de Boer et al., 2006; Hoffmann, 2001; Petrovici et al., 2005). For instance, Southern European countries exhibited a relatively high per capita supply of protein from vegetables and grains, whereas a relatively high supply of protein from milk was observed for Northern European countries (de Boer et al., 2006). Moreover, food trends, including flexitarian and vegan diets, in some population groups suggest that differences in the selection of protein sources are also present within countries (Aschemann-Witzel et al., 2021).

Protein source foods are central to the diet and are strongly associated with other foods in dietary patterns (De Gavelle et al., 2018). These patterns do not only display variations in protein levels, but also in the supply of additional nutrients included in the “protein package” (De Gavelle et al., 2018; Mariotti, 2019). Animal-based protein sources, such as meat, tend to be accompanied by zinc, vitamin B12, phosphorus and iron, whereas plant-based protein sources contribute more to intakes of fiber, vitamin E and magnesium (Phillips et al., 2015). Besides, contrasting environmental impacts have been observed according to patterns of protein consumption, with ruminant meat patterns expressing the highest overall environmental impacts and low meat patterns the lowest (Perraud et al., 2023). It may therefore be expected that different dietary protein patterns require distinct approaches for closing nutritional gaps and overcoming environmental challenges. A thorough understanding of protein consumption patterns is thus essential to successfully design and transition towards more sustainable diets in Europe.

Some studies have investigated individual-level dietary protein patterns within countries (De Gavelle et al., 2018; Perraud et al., 2023; Van Mierlo et al., 2021; Toujgani et al., 2023), whereas only one study explored this on a continental level in Europe using per capita consumption data from food balance sheets (de Boer et al., 2006). While covering a large geographical region is beneficial for comparisons between countries, a disadvantage to this approach is that it overlooks differences between demographic groups within countries as well as similarities between groups across countries that may require distinct or comparable dietary changes. Combining the strengths of both approaches, this study used individual-level food consumption data from 25 countries to identify unique dietary protein patterns in the European adult population and to assess potential differences in nutritional quality and environmental sustainability.

## 2 Methods

### 2.1 Study population and dietary data

Individual-level food consumption data were obtained from the most recent nationally-representative dietary surveys, available from the European Food Safety Authority (EFSA) Comprehensive European Food Consumption Database (European Food Safety Authority, 2022). This database is part of the EU Menu project that aims to increase the accessibility of high-quality, representative, detailed and harmonized food consumption data across Europe (European Food Safety Authority, 2014). From the 29 European countries included in the database, 25 countries for which dietary information of at least two non-consecutive consumption days for the adult population was available, were selected for our study (Supplemental Table 1).

Detailed information concerning the methodologies and protocols used for the dietary surveys can be retrieved from the original publications of each country, accessible through the EFSA database (European Food Safety Authority, 2022). In short, individual-level food consumption data were collected by all participating countries, of which 18 countries followed the standardized EU Menu methodology (European Food Safety Authority, 2014), and shared with EFSA. Data were obtained between 2003 and 2021 for 2–7 days by means of (web-based) food records and/or 24-/48-h dietary recalls (Supplemental Table 1). All food items were classified according to the FoodEx2 classification system developed by EFSA (European Food Safety Authority, 2015). We requested and received the food consumption data for research purposes via EFSA with permission from the countries. Among these were the new dietary surveys from the Balkan region (Gurinović et al., 2022), including Bosnia and Herzegovina (Arar et al., 2021), Montenegro (Martinovic et al., 2022) and Serbia (Zekovic et al., 2022).

Data were available for a total of 75,223 participants. Since some countries did not include younger and/or older age categories in their dietary surveys, we restricted the present study to the adult population aged 18–64 years (n = 31,536 excluded). Lactating (n = 280) or pregnant (n = 1105) participants were also excluded. Participants that reported only one consumption day (n = 1149) were left out as it does not allow for correction of within-person variation. Finally, implausible energy intakes (n = 1052), defined as participants in the lowest and highest 1.25% of reported energy intakes in each country separately, were excluded. In total 40,101 participants remained for the analysis.

### 2.2 Linkage with nutrient composition database

Since there is no standardized European-wide food composition database available, we used the Dutch Food Composition Database (NEVO version 2021/7.1) (Rijksinstituut voor Volksgezondheid en Milieu, 2021) to link food consumption data with detailed information on nutrient composition per food item. The Dutch database was selected due to its high quality and comprehensive inclusion of macro- and micronutrients. Two previously made linkages between FoodEx2 and NEVO (Mertens and Peñalvo, 2023; Mertens et al., 2022; van Rossum et al., 2020) were combined and evaluated, and extended by Vellinga et al. (2024). This provided information on nutrient composition of 2086 food items. Following the same methodology, 142 remaining food items (6% of food items, 0.8% of amount consumed) were matched to the NEVO code that most closely resembled the seventh level of the FoodEx2 classification based on nutritional value and/or ingredient composition. Artificial sweeteners, extracts and additives, food supplements, and foods for particular diets (2% of food items, 0.02% of amount consumed) could not be linked and were assumed to have no nutritional value. All coding was done by two researchers (M.C.D. and R.V.) and checked by a research dietician (J.D). It should be noted that some food items in the NEVO database are fortified with vitamins and/or minerals, such as certain milk products, fat spreads, meat and dairy imitates, and cereal products. Since specific information on fortification of these food items in the included European countries is scarce, we assumed that food fortification practices in Europe are similar to the Dutch market. This flaw was taken into consideration when interpreting the results.

### 2.3 Nutritional adequacy and quality

Nutritional adequacy of the diets was assessed by comparing nutrient intakes to a set of dietary reference values (DRVs) relevant for the European population. Daily nutrient intakes were calculated for each participant and standardized to a reference energy intake of 2000 kcal for females and 2500 kcal for males. Energy-standardized intakes were then expressed as a percentage of DRV fulfilment for each nutrient. DRVs were obtained from EFSA (European Food Safety Authority, 2017), the Nordic Nutrition Recommendations (Blomhoff et al., 2023), the WHO, WHO and Food and Agriculture Organization (2010), as detailed in Supplemental Table 2. For nutrients with an average requirement (AR) and population reference intake (PRI) available as DRVs, nutritional adequacy was calculated and presented for both, with the PRI as the primary outcome.

Overall nutritional quality of the diets was evaluated with the Nutrient Rich Diet (NRD) score (Van Kernebeek et al., 2014; Röös et al., 2015), derived from the Nutrient Rich Foods (NRF) index (Fulgoni et al., 2009; Drewnowski, 2009). The NRD algorithm was calculated as the unweighted sum of percentage DRVs for nutrients to encourage (i.e. qualifying nutrients) minus the sum of percentage DRVs for nutrients to limit (i.e. disqualifying nutrients). Qualifying nutrients were capped at 100%. In the present study, the NRD15.3 was used to capture as many nutrients that are potentially relevant to the European population. The NRD15.3 includes fifteen qualifying nutrients (protein, fiber, monounsaturated fatty acids (MUFA), vitamins A, B12, B1, B2, C, D and E, folate, calcium, iron, potassium and zinc) and three disqualifying nutrients (sugar, saturated fatty acids (SFA) and sodium).

### 2.4 Environmental sustainability

Diet-related environmental impacts were calculated using the SHARP-ID database (Mertens et al., 2019), which includes estimates of European average greenhouse gas emissions (GHGE) and land use (LU) of food items. In short, attributional life cycle assessment (LCA) was applied to quantify the environmental impacts throughout the entire life cycle of a food product, including primary production, primary packaging, transport, food losses and waste, and food preparations at home. Due to limited availability of data, industrial food processing, storage, and transport from retail to home were not included. To divide environmental impact between a product and its co-products, economic allocation was used for all foods, except for animal-sourced foods where nitrogen allocation was applied. LCA data were adjusted for consumption amount using available conversion factors for production, edible portion, cooking losses and gains, and food losses and waste. LCA data were available for 945 FoodEx2 coded foods, based on 182 primary food products, that are relevant to food consumption in four European countries (Denmark, Czechia, Italy and France).

To extend the LCA data to foods consumed in the remaining European countries, extrapolations were carried out for 1202 food items (53% of food items, 10% of amount consumed). Missing values were preferably supplemented with estimates for similar food items, comparable in production method and/or ingredient composition. Alternatively, the mean value of the same (and if not available higher) level of the FoodEx2 classification was used. For instance, *preserved tomatoes not concentrated* (level 4) was extrapolated with the mean value of all items in that specific subgroup, while *rice chips* (level 5) was extrapolated with the mean value of the higher subgroup *chips/crisps* (level 4). Furthermore, recipes were created for composite dishes based on a combination of food items if no suitable alternative was available. For instance, *beans, meat, and vegetables meal* was extrapolated with 1/3 *legumes based dishes*, 1/3 *mammals and birds meat*, and 1/3 *vegetables and vegetable products*. Spices, seasonings, extracts and additives, vinegar, mustard, food supplements, and foods for particular diets (6% of food items, 0.2% of amount consumed) could not be linked and were assumed to have no environmental impact. All extrapolations were done and checked by a team of two researchers (M.C.D. and S.B.).

Using the SHARP-ID and extrapolated data, daily GHGE (kg CO_2_-eq) and LU (m^2^∗year) were calculated and expressed in absolute and standardized amounts per 2000 kcal for each participant.

### 2.5 Socio-demographic and anthropometric characteristics

Information on age, sex, educational level, body weight and height was delivered by EFSA with permission from the countries. Age was categorized in three categories (18–34 years, 35–49 years and 50–64 years) and educational level was coded as low (no till lower secondary education), medium (upper secondary or post-secondary education) or high (university till post-university education). Body Mass Index (BMI) was calculated by dividing body weight by height squared (kg/m^2^) and participants were categorized as underweight, normal weight, overweight or obese based on BMI cut-off values of the World Health Organization (World Health Organization, 2010). Misreporting was identified using the Goldberg method (Goldberg et al., 1991), adopted by Black (2000), and participants were defined as under reporter, normal reporter or over reporter. Countries were assigned to one of the four European regions (i.e. West, East, North and South) based on the geographical classification of EuroVoc (European Union).

### 2.6 Statistical analysis

Dietary protein patterns were identified based on the consumption of protein source food groups. All FoodEx2 coded food items were grouped into 21 main- and 37 subgroups of foods and beverages (Supplemental Table 3). Food groups were considered a protein source when the main group had a weighted protein content of at least 5 g/100g or when the food group is promoted and consumed as a protein alternative (e.g. dairy imitates). A total of 24 protein source food groups were finally selected, which covered 88% of protein intake in the population (not accounting for protein intake from food products such as potatoes, vegetables, and sugar and confectionery). The amounts of foods consumed were summed within each of the selected food groups, averaged over the reported consumption days, and used as input for the following analyses.

To identify the dietary protein patterns, we applied a two-step procedure using principal component analysis (PCA) followed by hierarchical and K-means clustering, as proposed by Maugeri et al. (2022). The PCA was conducted on the covariance matrix of standardized (i.e. centred and scaled to unit variance) food group dietary data (Lever et al., 2017). This was done to reduce the size of the dataset while maintaining the variance and take away the noise of weak correlations. Multiple criteria were used to select the optimal number of principal components: breaks in the scree plot, eigenvalues >1, and a cumulative variance of >25% (Supplemental Fig. 1). The first six principal components (31.5% of total variance) were retained as input for the second step in which we applied hierarchical clustering using Ward's criterion to reveal different clusters based on the hierarchical tree (Ward, 1963). The optimal number of clusters was defined based on interpretation of the dendrogram construction and the elbow method (Supplemental Fig. 2). Finally, we selected six clusters, representing six distinct dietary protein patterns. The clustering solution was further consolidated using K-means clustering.

Multinomial logistic regression models were performed to assess the associations between socio-demographic and anthropometric characteristics (including sex, age, weight status and region of origin) and the identified dietary protein patterns. All variables were included in one model. Pairwise t-tests with Bonferroni correction were applied to test differences in food consumption, nutrient intakes, GHGE and LU between patterns. A two sided p-value of <0.05 was considered statistically significant.

All statistical analyses were conducted with R (version 4.2.1). PCA and clustering were performed using the *FactoMineR* package (Lê et al., 2008).

## 3 Results

### 3.1 Population characteristics

The study included a total of 40,101 participants, with a mean age of 42.0 years, of which 54.6% were female and 55.1% had overweight or obesity (Table 1). Underreporting of energy intake was relatively high in the study population, with 21.8% of participants classified as under reporter. The majority of the participants originated from Western Europe (47.3%), with 25.1% originating from Germany (Supplemental Table 4), followed by Eastern Europe (19.3%), Northern Europe (17.9%) and Southern Europe (15.4%).

**Table 1:** Socio-demographic and anthropometric characteristics of the total study population and the six dietary protein patterns, obtained from the EFSA Comprehensive European Food Consumption Database (European Food Safety Authority, 2022).

Characteristics^a^	Total (n = 40,101)		Common (n = 16,908, 42.2%)		Fast-food (n = 7828, 19.5%)		Milk-rich (n = 3946, 9.8%)		Health-conscious (n = 4832, 12.0%)		Traditional (n = 5942, 14.8%)		Plant-forward (n = 645, 1.6%)	
Sex														
Females	21,902	54.6%	11,835	70.0%	2527	32.3%	1945	49.3%	2590	53.6%	2591	43.6%	414	64.2%
Males	18,199	45.4%	5073	30.0%	5301	67.7%	2001	50.7%	2242	46.4%	3351	56.4%	231	35.8%
Age (years)^b^	42.0	13.1	42.7	13.1	42.2	13.0	38.9	13.4	43.4	13.2	40.9	12.9	39.8	12.3
Age category^b^														
18–34 years	12,199	31.8%	4790	29.7%	2299	30.6%	1553	40.6%	1376	29.0%	1950	35.2%	231	37.7%
35–49 years	13,528	35.3%	5740	35.6%	2701	36.0%	1315	34.4%	1591	33.5%	1960	35.3%	221	36.1%
50–64 years	12,648	33.0%	5604	34.7%	2506	33.4%	957	25.0%	1784	37.5%	1636	29.5%	161	26.3%
BMI (kg/m^2^)	25.9	4.7	25.9	4.7	26.4	4.5	25.9	4.7	25.9	4.8	25.6	4.5	24.5	4.5
Weight status^c^														
Underweight	714	1.8%	311	1.8%	92	1.2%	80	2.0%	74	1.5%	132	2.2%	25	3.9%
Normal weight	17,268	43.1%	7188	42.5%	2889	36.9%	1770	44.9%	2237	46.3%	2818	47.4%	366	56.7%
Overweight	15,809	39.4%	6723	39.8%	3522	45.0%	1466	37.2%	1743	36.1%	2166	36.5%	189	29.3%
Obese	6310	15.7%	2686	15.9%	1325	16.9%	630	16.0%	778	16.1%	826	13.9%	65	10.1%
Educational level^d^														
Unspecified	24,108	60.1%	10,050	59.4%	4706	60.1%	2692	68.2%	2656	55.0%	3680	61.9%	324	50.2%
Low	4060	10.1%	1727	10.2%	907	11.6%	380	9.6%	272	5.6%	727	12.2%	47	7.3%
Medium	5719	14.3%	2440	14.4%	1178	15.0%	448	11.4%	880	18.2%	693	11.7%	80	12.4%
High	6214	15.5%	2691	15.9%	1037	13.2%	426	10.8%	1024	21.2%	842	14.2%	194	30.1%
Under-/overreporting^b^^,^^e^														
Under reporter	8365	21.8%	5348	33.1%	866	11.5%	623	16.3%	750	15.8%	698	12.6%	80	13.1%
Normal reporter	29,544	77.0%	10,733	66.5%	6433	85.7%	3168	82.8%	3949	83.1%	4744	85.5%	517	84.3%
Over reporter	466	1.2%	53	0.3%	207	2.8%	34	0.9%	52	1.1%	104	1.9%	16	2.6%
Region^f^														
Western Europe	18,987	47.3%^g^	8737	46.0%^h^	4207	22.2%^h^	1947	10.3%^h^	2021	10.6%^h^	1721	9.1%^h^	354	1.9%^h^
Eastern Europe	7754	19.3%	2898	37.4%	2477	31.9%	349	4.5%	888	11.5%	1107	14.3%	35	0.5%
Northern Europe	7167	17.9%	2877	40.1%	722	10.1%	1057	14.7%	1755	24.5%	654	9.1%	102	1.4%
Southern Europe	6193	15.4%	2396	38.7%	422	6.8%	593	9.6%	168	2.7%	2460	39.7%	154	2.5%

*BMI* Body Mass Index, *SD* standard deviation.

a Continuous variables are presented as means with SDs and categorical variables are presented as counts with percentages.

b French population (n = 1726) not included in the calculations due to missing values for age.

c Based on BMI cut-off values of the World Health Organization (World Health Organization, 2010): underweight (<18.5), normal weight (18.5–24.9), overweight (25.0–29.9) and obese (≥30.0).

d Low (no till lower secondary education), medium (upper secondary or post-secondary education) and high (university till post-university education).

e Under-/overreporting of total energy intake estimated with the Goldberg method (Goldberg et al., 1991; Black, 2000).

f Based on geographical classification of EuroVoc (European Union): Northern Europe (Denmark, Estonia, Finland, Latvia and Sweden), Southern Europe (Cyprus, Greece, Italy, Portugal and Spain), Eastern Europe (Bosnia and Herzegovina, Croatia, Czechia, Hungary, Montenegro, Romania, Serbia and Slovenia) and Western Europe (Austria, Belgium, France, Germany, Ireland, Netherlands and United Kingdom).

g Percentage of the total number of participants in the study population.

h Percentage of the total number of participants in the respective region.

### 3.2 Dietary protein patterns

**Fig. 1:**
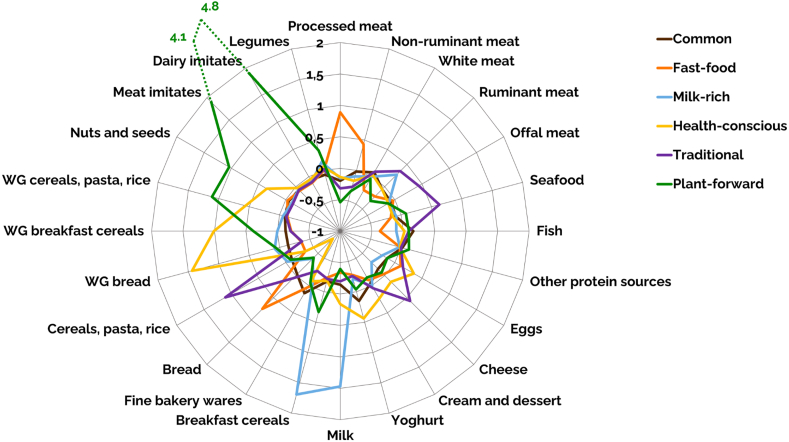
Comparison of mean z-scores of protein source food group consumption (g/2000 kcal) between the six dietary protein patterns, obtained from the EFSA Comprehensive European Food Consumption Database (European Food Safety Authority, 2022). A positive z-score indicates consumption above the mean of the total population, while a negative z-score indicates consumption below the mean. The magnitude of the z-score quantifies how many standard deviations consumption is away from the mean. For example, a z-score of 2 means that consumption is 2 standard deviations above the mean, while a z-score of −1 means the consumption is 1 standard deviation below the mean. *WG* wholegrain.

Six dietary protein patterns were identified based on the consumption of 24 protein source food groups (Supplemental Table 5). Fig. 1 displays the consumption profile of protein source food groups for each pattern relative to the total population. Most of the participants (42.2%) followed a *Common* protein pattern in which consumption aligned closely with the average consumption of protein source food groups in the population. In addition, they reported slightly higher intakes of fine bakery wares (+17% compared to the population average), sugar and confectionary (+12%), hot beverages (+22%), and sweetened beverages (+14%). The *Fast-food* protein pattern (19.5%) was characterized by a greater consumption of processed meat (+96%), non-ruminant meat (+83%) and refined bread (+51%), as well as the non-protein sources animal fats (+38%) and alcoholic beverages (+39%), whereas the consumption of fruit (−38%) and vegetables (−25%) were lower compared to all the other patterns. Participants belonging to the *Milk-rich* protein pattern (9.8%) consumed higher amounts of milk (+188%) and refined breakfast cereals (+592%). The *Health-conscious* protein pattern (12.0%) presented a higher consumption of yoghurt (+77%), eggs (+57%), cream and dessert (+48%), and wholegrain products, including wholegrain breakfast cereals (+360%) and wholegrain bread (+293%). We observed a *Traditional* protein pattern (14.8%) that included a diversity of meat products, such as offal meat (+228%) and ruminant meat (+54%), seafood (+317%), cheese (+62%), and refined cereals, pasta and rice (+131%). This pattern was also characterized by a higher consumption of vegetable oils and fats (+35%). A small proportion of the population (1.6%) could be identified as *Plant-forward*, consuming higher amounts of plant-based foods, such as meat imitates (+4514%), dairy imitates (+3966%), wholegrain cereals, pasta and rice (+536%), nuts and seeds (+317%), and legumes (+86%). The consumption of the non-protein sources vegetables (+37%) and fruit (+26%) was higher compared to all the other patterns.

### 3.3 Socio-demographic and anthropometric determinants

**Fig. 2:**
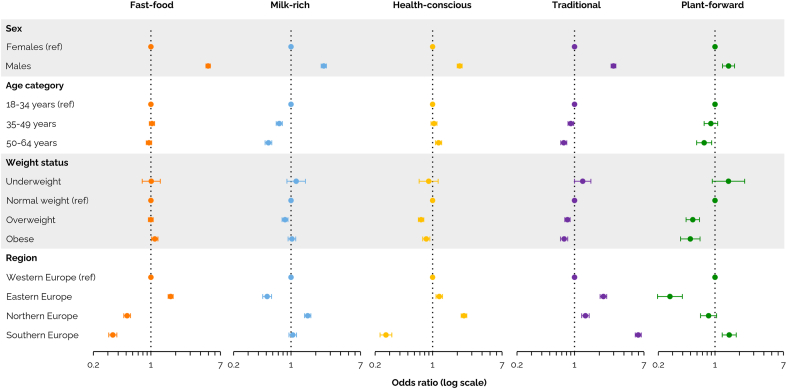
Associations between socio-demographic and anthropometric characteristics and the six dietary protein patterns, obtained from the EFSA Comprehensive European Food Consumption Database (European Food Safety Authority, 2022). Multinomial logistic regression models were adjusted for all variables presented in this figure. The *Common* protein pattern was used as a reference. Error bars represent 95% confidence intervals. *Ref* reference.

Fig. 2 (Supplemental Table 6) shows the associations between socio-demographic and anthropometric characteristics and the identified dietary protein patterns, with the *Common* protein pattern as the reference. Females were most likely to belong to the *Common* and *Plant-forward* protein patterns, with proportions of 70.0% and 64.2%, respectively, whereas males more often belonged to the *Fast-food* protein pattern (67.7%) (Table 1). Although age did not differ considerably between patterns, younger participants were most likely to adhere to the *Milk-rich* protein pattern and older participants more often followed the *Health-conscious* protein pattern. BMI was constant across most patterns, except for the *Fast-food* and *Plant-forward* protein patterns, where a respectively higher (26.4 kg/m^2^) and lower (24.5 kg/m^2^) BMI was observed. Correspondingly, participants with obesity were most likely to belong to the *Fast-food* protein pattern and least likely to belong to the *Plant-forward* protein pattern.

**Fig. 3:**
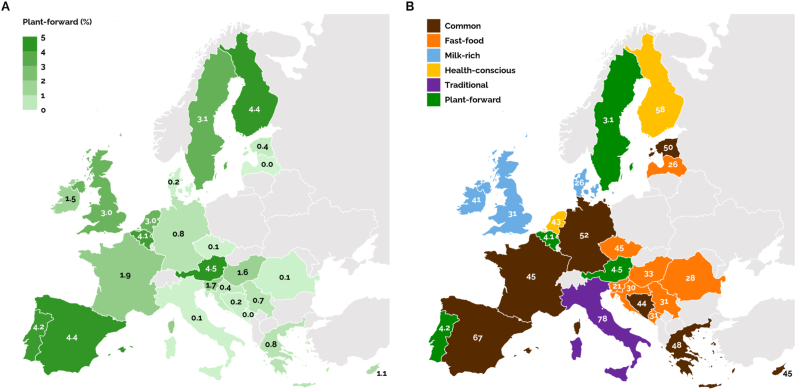
Occurrence of the six dietary protein patterns, obtained from the EFSA Comprehensive European Food Consumption Database (European Food Safety Authority, 2022), in 25 European countries, showing (A) the prevalence of the *Plant-forward* protein pattern and (B) the most prevalent pattern relative to all other countries. The latter was identified by applying min-max scaling to the prevalences of each pattern (columns) in Supplemental Table 4, and selecting the pattern with the highest scaled prevalence for each country (rows). Numbers represent prevalence percentages.

The *Common* protein pattern was the most occurring pattern in 19 out of 25 countries, with overall prevalences ranging from 16.1% in Italy to 66.7% in Spain (Supplemental Table 4). Although the *Plant-forward* protein pattern was least prevalent overall, this pattern was most commonly consumed in Austria, Finland, Spain, Portugal and Belgium, with prevalences ranging from 4.1% to 4.5% in these countries (Fig. 3A and Supplemental Table 4). Fig. 3B shows the most prevalent pattern in each country relative to all other countries, which was identified by applying min-max scaling to the prevalences of each pattern (columns) in Supplemental Table 4, and selecting the pattern with the highest scaled prevalence for each country (rows). The *Fast-food* protein pattern was predominantly found in Eastern European countries, with the highest prevalence observed for Czechia at 44.6%. Distinct preferences for other dietary protein patterns were evident across specific countries. The *Milk-rich* protein pattern was most frequently consumed in Ireland (40.6%), followed by the United Kingdom (31.1%) and Denmark (26.4%). The *Health-conscious* protein pattern was predominantly observed in Finland (57.6%) and the Netherlands (42.8%). Finally, Italy exhibited a high adherence to the *Traditional* protein pattern, with 77.9% of the population following this pattern.

### 3.4 Protein intake and sources

Protein intake was highest for the *Traditional* (93.6 g/day) and *Fast-food* (93.4 g/day) protein patterns and lowest for the *Common* protein pattern (63.8 g/day) (Supplemental Table 7). When accounting for energy intake, the *Traditional* protein pattern had the highest protein intake (17.4 E%), while the *Fast-food*, *Common* and *Plant-forward* protein patterns had the lowest (15.9–16.0 E%).

The proportion of animal (64–69%) and plant (31–36%) protein was similar across patterns, except for adherents to the *Plant-forward* protein pattern who consumed 52% animal and 48% plant protein. For all patterns, with the exception of the *Plant-forward* protein pattern, meat contributed most to the protein intake (30–42%), followed by either grains (21–26%) or dairy products (14–24%), and fish and seafood (2–12%). The *Plant-forward* protein pattern obtained most of its protein from grains (23%), meat (21%), dairy products (15%), fish and seafood (10%), and meat and dairy imitates (10%).

### 3.5 Nutritional adequacy and quality

**Fig. 4:**
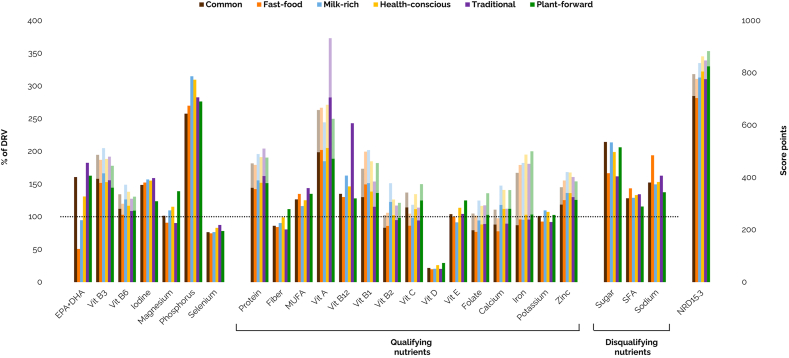
Energy-standardized nutrient intakes as a percentage of the dietary reference values (left axis) and Nutrient Rich Diet scores (right axis) of the six dietary protein patterns, obtained from the EFSA Comprehensive European Food Consumption Database (European Food Safety Authority, 2022). Transparent bars represent comparisons with the AR and non-transparent bars represent comparisons with the PRI where applicable. Nutrient intakes were standardized to a reference energy intake of 2000 kcal for females and 2500 kcal for males. DRVs were obtained from EFSA (European Food Safety Authority, 2017), the Nordic Nutrition Recommendations (Blomhoff et al., 2023), the WHO, WHO and Food and Agriculture Organization (2010). Qualifying and disqualifying nutrients were included in the calculation of the Nutrient Rich Diet score. *AR* average requirement, *DHA* docosahexaenoic acid, *DRV* dietary reference value, *EPA* eicosapentaenoic acid, *MUFA* monounsaturated fatty acids, *NRD* Nutrient Rich Diet, *PRI* population reference intake, *SFA* saturated fatty acids, *Vit* vitamin.

Energy intake varied across dietary protein patterns, with the lowest intake observed in the *Common* protein pattern (1643 kcal/day) and the highest in the *Fast-food* (2416 kcal/day) and *Traditional* (2191 kcal/day) protein patterns. Fig. 4 (Supplemental Table 8) shows the energy-standardized nutrient intakes as a percentage of the DRVs, as well as the NRD15.3 scores of the six dietary protein patterns. For all patterns, low intakes were observed for vitamin D (2.94–4.35 μg/day, 20–29% of DRV), potassium (3209–3835 mg/day, 92–110%) and selenium (52.1–61.1 μg/day, 74–87%), and intakes were high for protein (1.18–1.35 g/kg body weight, 142–162%), MUFA (11.6–14.4 E%, 116–144%), vitamin A (1289–1989 μg RE/day, 185–282%), vitamin B12 (5.12–9.71 μg/day, 128–243%), vitamin B1 (0.46–0.61 mg/1000 kcal, 115–151%), vitamin B3 (2.31–2.66 mg NE/MJ, 144–166%), vitamin B6 (1.72–2.09 mg/day, 103–126%), iodine (185–238 μg/day, 124–159%), phosphorus (1416–1730 mg/day, 258–315%) and zinc (9.56–11.6 mg/day, 119–136%). High intakes were observed for the disqualifying nutrients sugar (16.2–21.4 E%, 162–214%), SFA (11.6–14.3 E%, 115–143%) and sodium (2747–3874 mg/day, 137–194%).

Nutrients that were insufficient for some patterns were fiber, EPA+DHA, vitamins B2, C and E, folate, calcium, iron, and magnesium. Intakes of fiber (20.1–24.7 g/day, 80–99%), vitamin E (11.0–13.5 mg/day, 91–114%) and magnesium (296–372 mg/day, 90–115%) were low for all patterns, except for the *Plant-forward* protein pattern, with intakes of 27.8 g/day (111%), 14.6 mg/day (125%) and 441 mg/day (139%), respectively. EPA+DHA intakes were insufficient for the *Fast-food* (126 mg/day, 51%) and *Milk-rich* (236 mg/day, 95%) protein patterns and highest for the *Traditional* protein pattern (456 mg/day, 182%). Vitamin B2 (1.33–1.38 mg/day, 83–86%) and folate (254–262 μg DFE/day, 77–79%) intakes were particularly low for the *Common* and *Fast-food* protein patterns and highest for the *Milk-rich* (1.96 mg/day, 123%) and *Plant-forward* (339 μg DFE/day, 103%) protein patterns, respectively. Vitamin C intake was low for the *Fast-food* protein pattern (90.6 mg/day, 86%) and iron intake was low for the *Common* protein pattern (10.4 mg/day, 87%). Calcium intake was low for the *Fast-food* (748 mg/day, 78%), *Common* (844 mg/day, 88%) and *Traditional* (864 mg/day, 89%) protein patterns and similar for the other patterns (1069–1136 mg/day, 112–118%).

Overall, the *Fast-food* and *Common* protein patterns had the lowest nutritional quality, with NRD15.3 scores of 704 (−5%) and 712 (−4%) relative to the population average (Fig. 4 and Supplemental Table 8). In contrast, the *Plant-forward* and *Health-conscious* protein patterns achieved the highest nutritional quality, with scores of 825 (+11%) and 806 (+9%). Although both patterns had comparable contributions from qualifying nutrients to the score, the *Plant-forward* pattern achieved a higher overall score due to a lower penalty from intakes of disqualifying nutrients.

### 3.6 Environmental sustainability

**Fig. 5:**
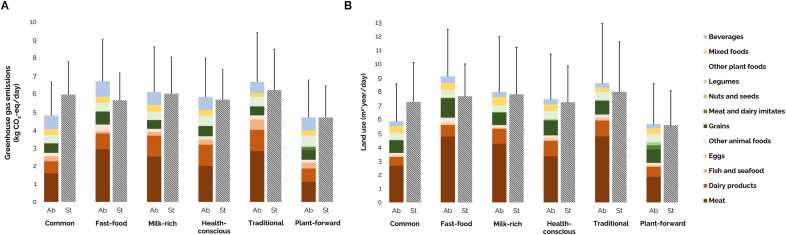
Contribution of food and beverage groups to the mean absolute (Ab) and energy-standardized (St) diet-related (A) greenhouse gas emissions and (B) land use of the six dietary protein patterns, obtained from the EFSA Comprehensive European Food Consumption Database (European Food Safety Authority, 2022). Standardized values are expressed per 2000 kcal. Error bars represent standard deviations. Other animal foods include other protein sources and animal fats. Other plant foods include starchy roots and tubers, vegetables, fruit, and vegetable oils and fats. Mixed foods include sugar and confectionary, composite dishes and miscellaneous. Beverages include hot, alcoholic and sweetened beverages and drinking water.

The contribution of food and beverage groups to the mean absolute and energy-standardized diet-related GHGE and LU of the six dietary protein patterns are presented in Fig. 5 (Supplemental Tables 9 and 10). Mean absolute GHGE ranged between 4.70 and 6.71 kg CO^2^-eq/day and mean absolute LU ranged between 5.66 and 9.11 m^2^∗year/day across patterns, with the lowest impacts for the *Plant-forward* and *Common* protein patterns. Differences in environmental impacts between all patterns, but the *Plant-forward* protein pattern, were smaller for energy-standardized values compared to absolute values. When accounting for energy intake, the highest impacts were observed for the *Traditional* protein pattern (GHGE: +5%, LU: +7%) and the lowest impacts were achieved by the *Plant-forward* protein pattern (GHGE: 20%, LU: 25%) compared to the population average. Impacts did not differ significantly between the other patterns.

Animal-based foods contributed more to environmental impacts (GHGE: 50–72%, LU: 51–74%) compared to plant-based foods (GHGE: 15–29%, LU: 18–37%), mixed foods (GHGE: 3–7%, LU: 4–9%) and beverages (GHGE: 9–16%, LU: 4–6%). For all patterns, meat was the main contributor to GHGE (24–44%) and LU (33–55%), but the degree varied widely across patterns. The *Fast-food*, *Milk-rich* and *Traditional* protein patterns reported the highest impacts from meat, which was mostly related to the consumption of processed, ruminant and non-ruminant meat. Dairy products, particularly cheese and milk (only for the *Milk-rich* protein pattern), were responsible for 13–20% and 9–15% of GHGE and LU, respectively. In contrast, grains contributed less to GHGE (7–11%) and slightly more to LU (12–17%), mainly due to the consumption of fine bakery wares and bread. Other major food groups’ contributions differed between patterns: fish and seafood (GHGE: 8%, LU: 1%) was relatively high for the *Traditional* protein pattern, vegetables (GHGE: 7%, LU: 4%) and meat and dairy imitates (GHGE: 4%, LU: 5%) were relatively high for the *Plant-forward* protein pattern, and hot beverages were relatively high for the *Common* (GHGE: 7%, LU: 3%) and *Plant-forward* (GHGE: 8%, LU: 2%) protein patterns.

## 4 Discussion

In this study, we identified six dietary protein patterns (i.e. *Common*, *Fast-food*, *Milk-rich*, *Health-conscious*, *Traditional* and *Plant-forward*) in the adult population from 25 European countries. More than 40% of the population consumed a *Common* protein pattern, with consumption closely aligning with the average consumption of protein source food groups, whereas 1.6% of the population (mainly from Austria, Finland, Spain, Portugal and Belgium) could be identified as *Plant-forward*. Average protein intake was sufficient in all patterns and largely came from animal products (64–69%), except for the *Plant-forward* protein pattern in which 52% of animal protein was consumed. Overall, the *Plant-forward* (+11%) and *Health-conscious* (+9%) protein patterns achieved the highest nutritional quality, reflected by the NRD15.3 score, whereas that of the *Fast-food* protein pattern was lowest (−5%) compared to the population average. GHGE and LU were comparable between patterns, except for the *Plant-forward* (GHGE: 20%, LU: 25%) and *Traditional* (GHGE: +5%, LU: +7%) protein patterns for which impacts were, respectively, lower and higher.

### 4.1 Identified dietary protein patterns

Although study populations differ, the identified dietary protein patterns in the current study are roughly comparable to patterns previously reported in Belgium (Van Mierlo et al., 2021) and France (De Gavelle et al., 2018; Perraud et al., 2023; Toujgani et al., 2023). Protein patterns that were frequently mentioned in these countries include a milk-based and fish-based pattern that resemble the *Milk-rich* and *Traditional* protein patterns. Other reported patterns differentiated by types of meat consumed (e.g. beef-based, pork-based and poultry-based); our European-wide dataset did not replicate these distinctive meat patterns, but identified the *Traditional* protein pattern that was high in ruminant meat, offal meat and seafood, and the *Fast-food* protein pattern that was high in processed and non-ruminant meat. Only in the NutriNet-Santé cohort of French volunteers, Toujgani et al. (2023) observed a healthy-plant-based protein pattern among 3% of the population, which was comparable to our *Plant-forward* protein pattern. Similarly, this pattern was characterized by a remarkably higher consumption of soy-based products (i.e. meat and dairy imitates), as well as higher amounts of legumes, nuts, fruit and vegetables. Unique in our study was the *Health-conscious* protein pattern, relatively high in whole grain products, yoghurt, fruit and vegetables, which was not reported in any of the other studies.

Our findings demonstrate significant country-level differences in the occurrence of dietary protein patterns across Europe. Previous research using per capita consumption data from food balance sheets – instead of individual-level daily consumption – highlighted the influence of geographic location on protein supply, showing that neighbouring countries often shared similar protein patterns (de Boer et al., 2006; Petrovici et al., 2005). The authors found that the Netherlands, Sweden and Finland were characterized by a high reliance on protein from milk, whereas Portugal, Italy and Greece favoured vegetables and cereals (de Boer et al., 2006). Consistent with these findings, we observed that the *Milk-rich* and *Health-conscious* protein patterns, respectively high in milk and yoghurt, were more prevalent in Northern and Western European countries than in other regions. In contrast, the relatively high consumption of plant foods, as seen in the *Plant-forward* protein pattern, was not restricted to a specific region and was most commonly found in Austria, Finland, Spain, Portugal and Belgium. Notably, a distinct dietary trend was observed in Eastern Europe, where the *Fast-food* protein pattern was predominantly prevalent.

In addition to these inter-country differences, there might be indications of a trend towards a more unified European diet (Hill, 1997; Naska et al., 2006; Dokova et al., 2022; Bajan et al., 2021). Our analyses revealed that most of the European adult population followed a pattern that was not very distinctive in the consumption of protein source food groups (i.e. *Common* protein pattern), which was also found in the Belgium population (Van Mierlo et al., 2021). Evidence suggests that Mediterranean countries, often characterized by traditional diets, have increasingly adopted more Westernized dietary habits over the past few decades (Naska et al., 2006; Bajan et al., 2021; Rumm-Kreuter, 2001). The consumption of protein-rich animal products, such as meat, eggs and dairy products, has increased in these countries (León-Muñoz et al., 2012; Martimianaki et al., 2022). Trends of dietary convergence are also evident along the East-West dimension of Europe, with studies indicating the adoption of healthier dietary behaviour (e.g. lower intakes of sugar, carbohydrates and SFA and higher consumption of fruit and vegetables) in Eastern European countries, following the processes that started in the 90s in many Western European countries (Dokova et al., 2022). Indeed, we observed that the *Health-conscious* protein pattern was also largely present in countries such as Slovenia, Serbia and Montenegro (Supplemental Table 4). Overall, these evolutions in dietary patterns have contributed to reducing the traditional differences between European diets, pointing towards a potential convergence in dietary habits across Europe.

### 4.2 Nutritional quality of the patterns

While some authors have expressed their concerns regarding the nutritional adequacy of more sustainable, plant-dominant diets (Beal et al., 2023; Fulgoni et al., 2024), our findings suggest that a balanced dietary shift from 65-70% to 50% animal protein is likely to sustain or even improve adequate nutrient levels across the general population. We found that the *Plant-forward* protein pattern, which obtained half of its protein from animal sources, achieved the highest nutritional quality and provided a diversity of nutrients. This is consistent with results from the NutriNet-Santé cohort study, which demonstrated that a healthy-plant-based protein pattern scored highest on the PANDiet score (i.e. nutritional adequacy score based on the probability of adequate nutrient intakes) and was associated with the lowest long-term health risk (Toujgani et al., 2023). Additionally, the *Plant-forward* protein pattern supplied high amounts of beneficial nutrients, including fiber, vitamins C and E, folate, and magnesium – nutrients that were lacking in other patterns and of which some are inadequate in the European population (Roman Viñas et al., 2011; Rippin et al., 2017). Notably, potential nutrient deficiencies commonly linked to plant-based diets, such as vitamin B12, calcium, iron, zinc and EPA+DHA, did not appear problematic in the *Plant-forward* protein pattern (Neufingerl and Eilander, 2022). Deficiencies in these nutrients are typically of concern when entire food groups, such as in vegetarian or vegan diets, are eliminated without proper diet planning. In this case, the *Plant-forward* protein pattern showed an above-average consumption of fish and seafood, which likely contributed to higher intakes of EPA+DHA and vitamin D compared to the other patterns. Recently, a French diet modelling study showed that a shift towards 30% animal protein with currently available foods would be possible while meeting nutrient requirements and maintaining health standards (Fouillet et al., 2023). Overall, a reduction in animal-based foods in the diet towards 50:50 and eventually 40:60 animal-to-plant protein, as proposed by some European countries (Vlaamse overheid, 2021; Raad voor de leefomgeving en infrastructuur, 2018), seems thus feasible and can provide a healthy diet for the general population (Heerschop et al., 2023).

Protein source foods unique to each dietary protein pattern seemed to play a role in the observed variations in nutrient intakes, highlighting their importance in shaping the overall nutritional profile of the diet. For instance, the *Fast-food* protein pattern expressed high intakes of SFA and sodium, probably related to the higher processed meat consumption; the *Milk-rich* protein pattern was characterized by a high intake of vitamin B2, which could be attributed to the higher milk consumption; and the *Traditional* protein pattern reported higher intakes of EPA+DHA and vitamins A and B12, likely due to the higher consumption of seafood and ruminant and offal meat. While similar conclusions were drawn by De Gavelle et al. (2018), the authors reported on other nutrients that were associated with the dietary protein patterns, probably due to slight differences in food consumption of the identified patterns in their study compared to ours. More advanced research techniques, such as network analysis, could help in understanding these complex links between food choices and nutrient intakes as well as other variables, such as demographics and environmental impacts, within dietary patterns (Epskamp and Fried, 2018).

In contrast to the variations in intakes of most nutrients, protein intake for this adult population was relatively stable and sufficient in all patterns. Average protein intake ranged between 1.18 and 1.35 g/kg body weight across patterns and remained well above the PRI of 0.83 g/kg body weight. Adequate intakes of protein were also reported in previous studies investigating dietary protein patterns (De Gavelle et al., 2018; Van Mierlo et al., 2021; Toujgani et al., 2023). Similar to these studies, the current study focused solely on protein quantity and did not account for the quality of the proteins in the different protein source foods. Research indicates that plant foods generally have a lower protein quality due to less favourable amino acid profiles and lower digestibility compared to animal products (Mariotti, 2017). As such, bioavailable protein may have been lower in the *Plant-forward* protein pattern due to the higher proportion of plant protein in the diet. Nevertheless, the diverse array of plant foods in this pattern can probably still provide adequate protein when sufficiently consumed and appropriately combined in meals or over the day (e.g. grains with legumes), as supported by research on the complementarity of plant proteins in meeting essential amino acid requirements (Mariotti, 2017; Hertzler et al., 2020; Adhikari et al., 2022).

### 4.3 Environmental sustainability of the patterns

Our research indicates that the environmental footprint of current diets may be more determined by the quantity of animal products consumed rather than the specific combination of these products in the diet. For instance, despite differences in (animal) protein consumption habits, the *Common*, *Fast-food*, *Milk-rich* and *Health-conscious* protein patterns maintained a similar animal-to-plant protein ratio and showed minimal variation in environmental impacts. In contrast, the *Plant-forward* protein pattern achieved significantly lower impacts, primarily due to the lower amount of animal protein in this pattern. Notably, patterns associated with a higher overall consumption of animal products, such as the *Fast-food* pattern, showed greater absolute environmental impacts due to the high amount of these high-impact foods in the diet. These findings align with a substantial body of literature reporting on the environmental benefits of both observed and modelled plant-based diets (Aleksandrowicz et al., 2016; Jarmul et al., 2020; Scarborough et al., 2023; Hallström et al., 2015). Additionally, it was recently demonstrated that the animal-to-plant protein ratio of diets is strongly associated with environmental impacts, including GHGE and LU (Fouillet et al., 2023). Reducing the overall consumption of animal-based foods and shifting dietary habits towards more plant-based foods is thus essential for mitigating environmental impacts and meeting climate goals.

Nonetheless, it should be noted that significant variations in environmental impacts exist between various types of animal products (Clark et al., 2019), as well as between different agricultural production systems (Poore and Nemecek, 2018). Previous studies especially emphasize the high impacts from red meat, in terms of GHGE, LU and nutrient pollution, whereas impacts of white meat and dairy products are estimated to be lower (Clark et al., 2019; Poore and Nemecek, 2018). In support of these findings, our analyses revealed that the *Traditional* protein pattern displayed the highest environmental impacts, largely due to the higher consumption of ruminant meat. These results are in accordance with two studies from France, which similarly reported higher GHGE and LU for both ruminant and total meat patterns, also showing poorer performance on other environmental indicators including cumulative energy demand, terrestrial eutrophication, acidification and particulate matter emissions (Perraud et al., 2023; Toujgani et al., 2023).

### 4.4 Strengths and limitations

Among the strengths of the present study is the inclusion of detailed and harmonized dietary information of the adult population from 25 European countries that allowed a thorough analysis of dietary protein patterns across diverse cultural settings within Europe. We specifically focused on protein consumption habits which are at the core of the current dietary debate surrounding health and environmental sustainability of food systems.

However, we used dietary data collected from 2003 to 2021, which may not fully reflect current dietary habits, especially given the increasing popularity of plant-based diets in Europe. Considering that about 30% of Europeans now identify themselves as flexitarian (Smart Protein, 2021), it can be expected that a larger proportion of the population would belong to the *Plant-forward* protein pattern today. Additionally, the prevalence of underreporting was relatively high in our study population and varied across dietary protein patterns (11.5–33.1%). Although energy standardization was applied to correct for this, the potential bias remains uncertain as it is unclear which (protein source) foods are more prone to underreporting (Ravelli and Schoeller, 2020). Furthermore, due to the lack of standardized and complete national food composition and environmental impact data, we relied on data specific to the Dutch and European contexts, respectively. While we assumed that food fortification practices in other European countries were similar to those in the Netherlands, it is expected that these practices vary across Europe (e.g. iodine fortification of salt), though specific information on this is limited (Westenbrink et al., 2024; Hennessy et al., 2013). We also did not account for the intake of food supplements, which could significantly contribute to nutritional adequacy (Blumberg et al., 2017). Finally, differences in environmental impacts may also exist between countries due to variations in production systems, import and export practices, and transport methods and distances (Notarnicola et al., 2017). While the scarcity of country-specific environmental impact data necessitated the use of European average data (Mertens et al., 2019; Alves et al., 2024), this enabled us to focus on differences related to food consumption rather than food production, aligning with our research objectives.

## 5 Conclusions

This study identified a diversity of dietary protein patterns in the adult population from 25 European countries that displayed unique nutritional profiles and varying environmental impacts. While the *Plant-forward* protein pattern showed that a lower consumption of animal-based protein foods and a higher consumption of plant-based protein foods can achieve both a higher nutritional quality and lower environmental impacts, most Europeans still rely heavily on animal proteins in their diet. To make significant progress in meeting health and climate goals, large efforts from policy are needed to guide the current consumption of animal products towards more plant foods. Our study shows that the *Plant-forward* protein pattern, which still obtained half of its protein from animal sources, could be a realistic and feasible example that aligns with these goals. Moving forward, targeted strategies that address the specific nutritional and environmental gaps of each identified dietary protein pattern, while considering the cultural and demographic context of individual countries, will be essential for fostering sustainable dietary transitions across Europe.

## CRediT authorship contribution statement

**Merel C. Daas:** Conceptualization, Methodology, Data curation, Formal analysis, Visualization, Writing – original draft, Writing – review & editing. **Pieter van 't Veer:** Conceptualization, Funding acquisition, Methodology, Writing – review & editing, Supervision. **Elisabeth H.M. Temme:** Methodology, Writing – review & editing. **Anneleen Kuijsten:** Methodology, Writing – review & editing. **Mirjana Gurinović:** Funding acquisition, Methodology, Writing – review & editing. **Sander Biesbroek:** Conceptualization, Funding acquisition, Methodology, Writing – review & editing, Supervision. All authors have read and agreed to the published version of the manuscript.

## Funding

This work received funding from the European Union's 10.13039/501100007601Horizon 2020 research and innovation program under grant agreement number 101059632 (project GIANT LEAPS).

## Declaration of competing interest

The authors declare that they have no known competing financial interests or personal relationships that could have appeared to influence the work reported in this paper.

## Data Availability

Individual-level food consumption data are available upon request from the European Food Safety Authority: https://data.europa.eu/data/datasets/the-efsa-comprehensive-european-food-consumption-database?locale=en. Other analyzed datasets are publicly available: https://nevo-online.rivm.nl/; https://doi.org/10.17026/dans-xvh-x9wz.
