# Supplementary material for: Diversity of dietary protein patterns across Europe – Impact on nutritional quality and environmental sustainability
Source: Curr Res Food Sci. 2025 Mar 4;10:101019. doi: 10.1016/j.crfs.2025.101019 (PMC11946498; doi:10.1016/j.crfs.2025.101019)
Supplement: Multimedia component 1 [file mmc1.pdf]

Supplementary information

Diversity of Dietary Protein Patterns Across Europe – Impact on Nutritional Quality and Environmental Sustainability

Merel C. Daas[a,*], Pieter van 't Veer[a], Elisabeth H.M. Temme[a,b], Anneleen Kuijsten[a], Mirjana Gurinović[c,d], Sander Biesbroek[a]

[a] Division of Human Nutrition and Health, Wageningen University & Research, 6700 AA Wageningen, The Netherlands

[b] Centre for Prevention, Lifestyle and Health, Department for Healthy and Sustainable Nutrition, National Institute for Public Health and the Environment (RIVM), 3721 MA Bilthoven, The Netherlands

[c] Centre of Research Excellence in Nutrition and Metabolism, Institute for Medical Research, National Institute of Republic of Serbia, University of Belgrade, 11000 Belgrade, Serbia

[d] Capacity Development in Nutrition (CAPNUTRA), Belgrade, Serbia

* Corresponding author. *Email address:* merel.daas@wur.nl (Merel C. Daas).

Supplemental Table 1 Details of the included national dietary surveys for the adult population available from the EFSA Comprehensive European Food Consumption Database [1].

Country	Survey name	Period	EU Menu method	Dietary assessment	Number of days	Age range	Original sample size	Included sample size
Austria	AT-NATIONAL-2016	2014-2018	Yes	24-hour dietary recall	2	18-64	2250	2106
Belgium	NATIONAL-FCS-2014	2014-2015	Yes	Food record, 24-hour dietary recall	2	3-64	3305	1170
Bosnia and Herzegovina	B&H MENU	2017-2020	Yes	24-hour dietary recall	2	10-64	1529	826
Croatia	NIPNOP-HAH-2011-2012	2011-2012	Yes	24-/48-hour dietary recall	3	18-64	2002	1929
Cyprus	CY 2014-2017-LOT2	2014-2017	Yes	24-hour dietary recall	3	10-76	1016	264
Czechia	SISP04	2003-2004	No	24-hour dietary recall	2	4-64	2353	1624
Denmark	DANSDA 2005-08	2005-2008	No	Food record	7	4-75	2700	1640
Estonia	DIET-2014-EST-A	2013-2015	Yes	24-hour dietary recall	2	11-75	3049	2028
Finland	FINDIET 2017	2017	Yes	24-hour dietary recall	2	18-75	1773	1142
France	INCA3	2014-2015	Yes	Food record, 24-hour dietary recall	3	0-79	4847	1726
Germany	NATIONAL NUTRITION SURVEY II	2007	No	24-hour dietary recall	2	14-80	13,926	10,071
Greece	GR-EFSA-LOT2 2014-2015	2014-2016	Yes	24-hour dietary recall	2	10-75	798	251
Hungary	EU MENU DIETARY SURVEY OF HUNGARY	2018-2020	Yes	Food record, 24-hour dietary recall	2	1-74	2689	504
Ireland	NANS 2012	2008-2010	No	Food record	4	18-90	1500	1242
Italy	INRAN SCAI 2005-06	2005-2006	No	24-hour dietary recall	3	0-97	3323	2226
Latvia	LATVIA_2014	2012-2015	Yes	Food record, 24-hour dietary recall	2	0-80	3595	1052
Montenegro	EUMENU ADLT	2017-2021	Yes	24-hour dietary recall	2	10-74	1513	679
Netherlands	FCS2016_CORE	2012-2016	Yes	Food record, 24-hour dietary recall	2	1-80	4313	1440
Portugal	IAN-AF 2015-2016	2015-2016	Yes	Food record, 24-hour dietary recall	2	0-84	6429	2930
Romania	RO-DIET-NATIONAL-STUDY-2019	2019-2020	Yes	24-hour dietary recall	2	10-74	1730	711
Serbia	RS_ADULTS	2017-2022	Yes	24-hour dietary recall	2	10-75	2737	1119
Slovenia	SI.MENU-2018	2017-2018	Yes	Food record, 24-hour dietary recall	2	0-74	1981	362
Spain	ENALIA2	2013-2015	Yes	24-hour dietary recall	2	18-74	968	522
Sweden	RIKSMATEN 2010	2010-2011	No	Web-based food record	4	18-80	1797	1305
United Kingdom	NDNS ROLLING PROGRAMME YEARS 1-3	2008-2011	No	Food record	4	1-94	3073	1232

Supplemental Table 2 Selection of nutrients to evaluate nutritional adequacy of the dietary protein patterns, including the used dietary reference values.

Nutrients	Type of DRV	DRV[1]	Source
**Other nutrients**			
EPA+DHA	AI	250 mg/day	European Food Safety Authority [2]
Vit B3	AR	1.3 mg NE/MJ	European Food Safety Authority [2]
	PRI	1.6 mg NE/MJ	
Vit B6	AR	1.5 / 1.3 mg/day	European Food Safety Authority [2]
	PRI	1.7 / 1.6 mg/day	
Iodine	AI	150 µg/day	European Food Safety Authority [2]
Magnesium	AI	350 / 300 mg/day	European Food Safety Authority [2]
Phosphorus	AI	550 mg/day	European Food Safety Authority [2]
Selenium	AI	70 µg/day	European Food Safety Authority [2]
**Qualifying nutrients**			
Protein	AR	0.66 g/kg BW	European Food Safety Authority [2]
	PRI	0.83 g/kg BW	
Fiber	AI	25 g/day	European Food Safety Authority [2]
MUFA	RI	10 E%	Nordic Nutrition Recommendations [3]
Vit A	AR	570 / 490 µg RE/day	European Food Safety Authority [2]
	PRI	750 / 650 µg RE/day	
Vit B12	AI	4 µg/day	European Food Safety Authority [2]
Vit B1	AR	0.3 mg/1000 kcal	European Food Safety Authority [2]
	PRI	0.4 mg/1000 kcal	
Vit B2	AR	1.3 mg/day	European Food Safety Authority [2]
	PRI	1.6 mg/day	
Vit C	AR	90 / 80 mg/day	European Food Safety Authority [2]
	PRI	110 / 95 mg/day	
Vit D	AI	15 µg/day	European Food Safety Authority [2]
Vit E	AI	13 / 11 mg/day	European Food Safety Authority [2]
Folate	AR	250 µg DFE/day	European Food Safety Authority [2]
	PRI	330 µg DFE/day	
Calcium	AR	750 / 860 mg/day[2]	European Food Safety Authority [2]
	PRI	950 / 1000 mg/day[2]	
Iron	AR	6 / 7 mg/day[3]	European Food Safety Authority [2]
	PRI	11 / 16 mg/day[3]	
Potassium	AI	3500 mg/day	European Food Safety Authority [2]
Zinc	AR	7.5 / 6.2 mg/day	European Food Safety Authority [2]
	PRI	9.4 / 7.5 mg/day	
**Disqualifying nutrients**			
Sugar	UL	10 E%	World Health Organization [4]
SFA	UL	10 E%	Food and Agriculture Organization [5]
Sodium	UL	2000 mg/day	World Health Organization [6]

AI adequate intake, *AR* average requirement, *BW* body weight, *DFE* dietary folate equivalents, *DRV* dietary reference value, *E%* energy percentage, *MUFA* monounsaturated fatty acids, *PRI* population reference intake, *RE* retinol equivalents, *RI* reference intake, *SFA* saturated fatty acids, *UL* upper limit, *Vit* vitamin.

[1] Set for both sexes or males/females separately (unless otherwise specified).

[2] 860 mg/day and 1000 mg/day for individuals under 25 years.

[3] 7 mg/day and 16 mg/day for females under 40 years.

Supplemental Table 3 Selection of protein source food groups for the identification of dietary protein patterns.

Food group	Description	Protein content (g/100 g)	Weighted protein content (g/100 g)	Protein source[1]
**Animal-based foods**				
Meat		18.0	20.1	
Processed meat	Dried meat, marinated meat, canned-tinned meat, cured meat, preserved fat tissue, animal mechanically separated meat (MSM), sausages, meat burger, meat balls, meat loaf, meat terrine, pate	16.8	16.8	X
Red meat		21.6	21.1	
Ruminant meat	Beef, goat, sheep, deer fresh meat	21.4	20.8	X
Non-ruminant meat	Pork, horse fresh meat	22.0	21.5	X
White meat	Poultry, rabbit fresh meat	22.7	23.1	X
Offal meat	Fresh fat tissue, liver, kidney, tongue, heart, gelatine	16.7	15.9	X
Fish and seafood		22.6	23.6	
Fish	Fish meat, fish liver, fish roe, dried fish, smoked fish, fishcakes, fish pate, canned/jarred fish, fish paste, marinated fish, salt-preserved fish	23.3	24.1	X
Seafood	Crustaceans, molluscs, sea urchins, canned seafood, salted seafood, smoked seafood, marinated seafood, dried seafood	20.2	20.5	X
Other protein sources	Amphibians, reptiles, snails, insects	18.1	17.3	X
Eggs	Whole egg, egg powder, egg yolk, egg white, boiled egg, fried egg, poached egg, omelette	14.6	12.2	X
Dairy products		17.1	6.0	
Milk	Plain milk, buttermilk, fermented milk, flavoured milk, evaporated milk, condensed milk, milk powder, whey, whey powder	8.5	3.5	X
Yoghurt	Plain yoghurt, flavoured yoghurt, yoghurt drink, quark, skyr	4.5	3.8	X
Cream and dessert	Cream, sour cream, creme fraiche, cream powder, custard, starchy pudding, dairy ice cream, frozen yoghurt, milkshake, dairy snacks	4.1	2.8	X
Cheese	Fresh uncured cheese, ripened cheese, feta, cheese powder, cheese wedges, cheese spreads	22.7	21.1	X
Animal fats	Pork lard, butter, ghee, fish oil	0.3	0.6	
**Plant-based foods**				
Grains		7.8	8.1	
Refined grains		7.3	8.2	
Breakfast cereals	Cereal flakes, cereal bars, extruded breakfast cereal products	8.8	9.0	X
Fine bakery wares	Biscuits, cakes, beignets, croissant, doughnut, flan tart, marzipan pie, apple strudel, baklava, pancakes, scones, waffles, macaroons, sweet doughs	6.0	6.1	X
Bread	Refined bread, gluten-free bread, pretzels, crackers, breadsticks, refined crisp bread, puffed bread, extruded bread, refined rusk, pita bread, tortilla, pizza base, croutons, bread doughs	9.5	10.0	X
Cereals, pasta, rice	Refined flour, semolina, white rice, fresh pasta, dried pasta, gluten-free pasta, noodles, couscous, gnocchi, crisps	7.2	5.8	X
Whole grains		9.2	7.7	
WG breakfast cereals	Rolled grains, popped cereals, muesli, porridge	6.7	6.5	X
WG bread	Wholegrain bread, pumpernickel, wholegrain crisp bread, wholegrain rusk	9.7	8.5	X
WG cereals, pasta, rice	Cereal grains, cereal bran, cereal germ, groats, wholegrain flour, brown rice, wholegrain pasta	10.8	7.0	X

Starchy roots and tubers	Potatoes, cassava, sweet potatoes, sugar beet roots, potato puree, potato flakes, mashed potato powder, potato flour, fries, baked potato, potato crisps, potato snacks	2.5	2.0	
Vegetables	Broccoli, cauliflower, cabbage, Brussels sprouts, kale, garlic, onion, tomatoes, peppers, aubergine, cucumber, courgette, pumpkin, corn, lettuce, spinach, beetroot, carrots, radishes, asparagus, sprouts, fungi, legumes with pod, algae, herbs, dried vegetables, canned/jarred vegetables, fermented vegetables, vegetable puree, grilled vegetables, boiled vegetables, fried vegetables, mixed green salad, pickles	2.3	1.3	
Fruit	Citrus fruits, apples, pears, berries, apricots, cherries, peaches, kumquats, kiwi, bananas, mango, pineapple, dates, figs, olives, avocado, dried fruit, canned/jarred fruit, fruit salad, fruit puree	0.9	0.6	
Legumes	Fresh legumes, canned/jarred legumes, dried pulses, pulses flour, hummus	10.4	7.0	X
Nuts and seeds	Almonds, cashew nuts, hazelnuts, walnuts, linseed, peanuts, sunflower seeds, chia seeds, mustard, sesame paste, peanut butter	19.3	20.7	X
Vegetable oils and fats	Olive oil, rape seed oil, sunflower oil, coconut oil, cocoa butter, margarine	0.1	0.1	
Meat and dairy imitates		5.3	3.9	
Meat imitates	(Fermented) soyabean-based meat imitates, fungi-based meat imitates, tofu	13.0	12.6	X
Dairy imitates	Soya drink, almond drink, oat drink, imitation cream, non-dairy coffee creamer, soya yoghurt, imitation cheese	2.4	2.5	X
**Mixed foods**				
Sugar and confectionary	Jam, marmalade, compote, chutney, candied fruits, sugar, honey, molasses, syrup, sweeteners, chocolate, candies, marzipan, caramel, nougat, sorbet	2.3	2.0	
Composite dishes	Soup, salad, Shepherd’s pie, goulash, fish gratin, vegetable casserole, quiche, sandwich, hot dog, pizza, spring rolls, stuffed pasta, risotto, paella	6.3	4.5	
Miscellaneous	Starches, spices, seasoning, sauces, vinegar, salad dressing, stock cubes, food flavourings, food colours, thickener, flavour enhancer, isolated proteins, food for non-standard diets, food supplements	7.0	3.4	
**Beverages**				
Hot beverages	Coffee, tea, herbal infusions, cocoa beverages	2.2	0.2	
Alcoholic beverages	Beer, wine, liquors, spirits, mixed alcoholic drinks	0.3	0.3	
Sweetened beverages	Fruit juices, vegetable juices, soft drinks, energy drinks, coconut water	0.3	0.2	
Drinking water	Tap water, bottled water, flavoured water	0.0	0.0	

[1] Food groups were considered a protein source (X) when the main food group had a weighted protein content of at least 5 g/100g or when the food group is promoted and consumed as a protein alternative (e.g. dairy imitates).

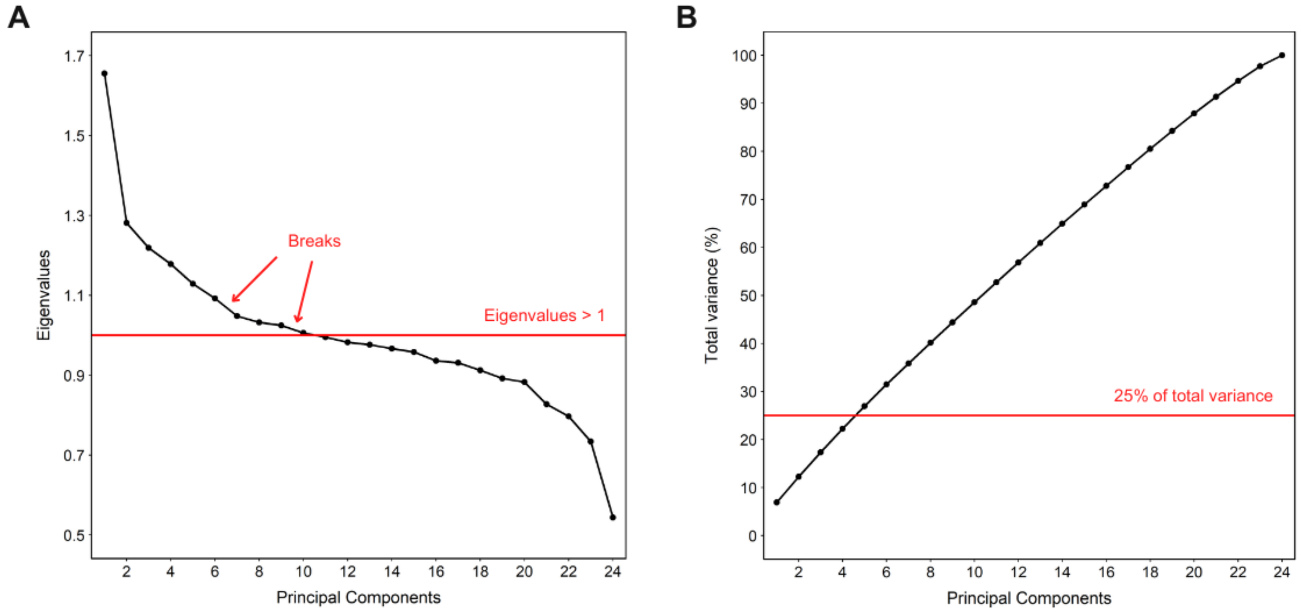

Supplemental Figure 1 Approaches used to select the optimal number of principal components: (A) breaks in the scree plot and eigenvalues > 1 and (B) cumulative variance of > 25%.

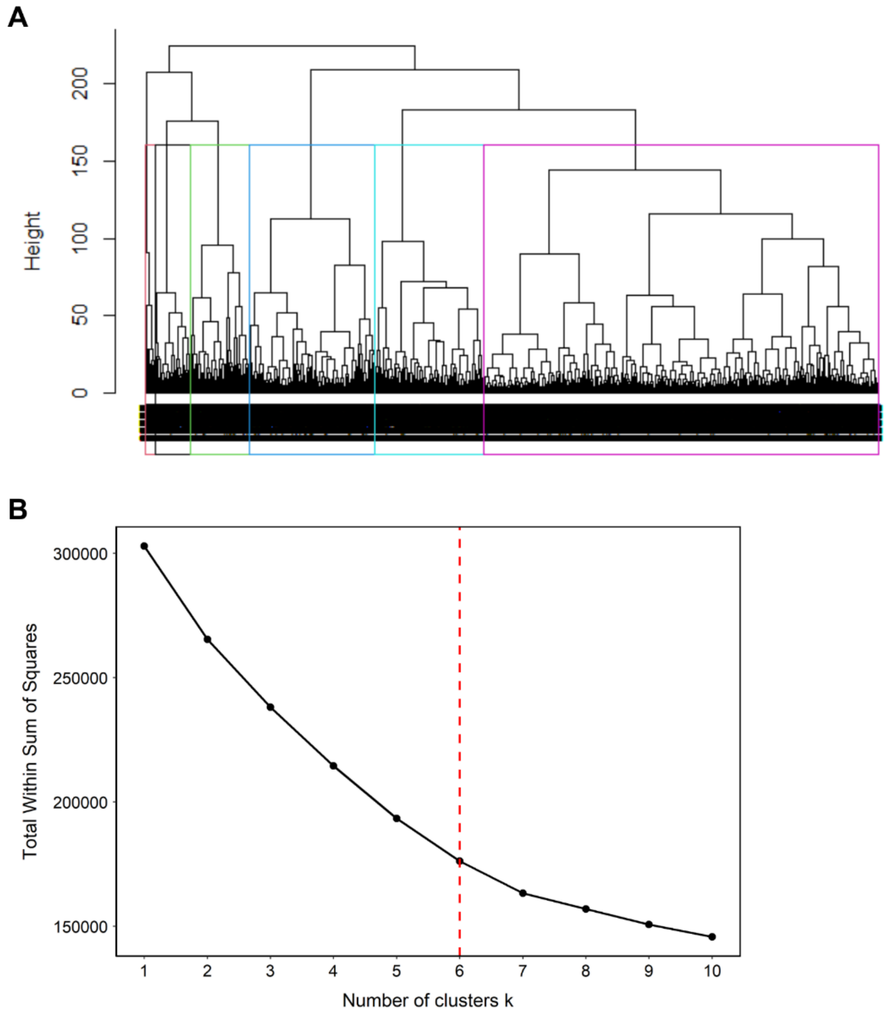

Supplemental Figure 2 Approaches used to select the optimal number of clusters: (A) interpretability of the dendrogram construction and (B) elbow method.

Supplemental Table 4 Country characteristics of the total study population and the six dietary protein patterns, obtained from the EFSA Comprehensive European Food Consumption Database [1].

	Total (n = 40,101)		Common (n = 16,908, 42.2%)		Fast-food (n = 7828, 19.5%)		Milk-rich (n = 3946, 9.8%)		Health-conscious (n = 4832, 12.0%)		Traditional (n = 5942, 14.8%)		Plant-forward (n = 645, 1.6%)	
**Country**	n	%[1]	n	%[2]	n	%[2]	n	%[2]	n	%[2]	n	%[2]	n	%[2]
Austria	2106	5.3	909	43.2	460	21.8	104	4.9	222	10.5	316	15.0	95	4.5
Belgium	1170	2.9	558	47.7	160	13.7	87	7.4	197	16.8	120	10.3	48	4.1
Bosnia and Herzegovina	826	2.1	367	44.4	181	21.9	16	1.9	150	18.2	110	13.3	2	0.2
Croatia	1929	4.8	676	35.0	573	29.7	140	7.3	98	5.1	434	22.5	8	0.4
Cyprus	264	0.7	118	44.7	47	17.8	52	19.7	15	5.7	29	11.0	3	1.1
Czechia	1624	4	606	37.3	724	44.6	59	3.6	9	0.6	224	13.8	2	0.1
Denmark	1640	4.1	647	39.5	126	7.7	433	26.4	327	19.9	104	6.3	3	0.2
Estonia	2028	5.1	1010	49.8	245	12.1	204	10.1	242	11.9	319	15.7	8	0.4
Finland	1142	2.8	206	18.0	28	2.5	118	10.3	658	57.6	82	7.2	50	4.4
France	1726	4.3	774	44.8	322	18.7	121	7.0	81	4.7	396	22.9	32	1.9
Germany	10,071	25.1	5238	52.0	2936	29.2	645	6.4	511	5.1	661	6.6	80	0.8
Greece	251	0.6	120	47.8	33	13.1	34	13.5	13	5.2	49	19.5	2	0.8
Hungary	504	1.3	126	25.0	168	33.3	59	11.7	82	16.3	61	12.1	8	1.6
Ireland	1242	3.1	307	24.7	94	7.6	504	40.6	285	22.9	33	2.7	19	1.5
Italy	2226	5.6	358	16.1	62	2.8	42	1.9	27	1.2	1734	77.9	3	0.1
Latvia	1052	2.6	461	43.8	277	26.3	85	8.1	161	15.3	68	6.5	0	0.0
Montenegro	679	1.7	272	40.1	208	30.6	12	1.8	136	20.0	51	7.5	0	0.0
Netherlands	1440	3.6	444	30.8	169	11.7	103	7.2	616	42.8	65	4.5	43	3.0
Portugal	2930	7.3	1452	49.6	248	8.5	399	13.6	103	3.5	605	20.6	123	4.2
Romania	711	1.8	310	43.6	197	27.7	30	4.2	69	9.7	104	14.6	1	0.1
Serbia	1119	2.8	406	36.3	349	31.2	14	1.3	259	23.1	83	7.4	8	0.7
Slovenia	362	0.9	135	37.3	77	21.3	19	5.2	85	23.5	40	11.0	6	1.7
Spain	522	1.3	348	66.7	32	6.1	66	12.6	10	1.9	43	8.2	23	4.4
Sweden	1305	3.3	553	42.4	46	3.5	217	16.6	367	28.1	81	6.2	41	3.1
United Kingdom	1232	3.1	507	41.2	66	5.4	383	31.1	109	8.8	130	10.6	37	3.0

[1] Percentage of the total number of participants in the study population.

[2] Percentage of the total number of participants in the respective country.

Supplemental Table 5 Energy-standardized[1] consumption of food groups of the six dietary protein patterns, obtained from the EFSA Comprehensive European Food Consumption Database [1].

	Total			Common			Fast-food			Milk-rich			Health-conscious			Traditional			Plant-forward		
	Mean	Mdn	IQR	Mean	Mdn	IQR	Mean	Mdn	IQR	Mean	Mdn	IQR	Mean	Mdn	IQR	Mean	Mdn	IQR	Mean	Mdn	IQR
**Animal-based foods**																					
Meat[2]	143	127	74.1, 192	133*	116	63.4, 182	178*	166	112, 230	143	130	78.5, 191	125*	113	65.3, 170	144	125	76.4, 187	79.8*	51.9	0.0, 121
Processed meat	44.8	30.9	8.1, 65.2	35.0	23.9	0.0, 52.3	87.9*	76.7	43.6, 120	36.9	26.5	7.1, 54.5	38.2	26.3	4.9, 57.1	29.1*	21.4	4.1, 44.0	18.8*	0.0	0.0, 26.7
Red meat	58.2	41.2	0.0, 88.0	54.4*	35.7	0.0, 84.5	66.8	49.6	0.0, 99.4	68.0	53.0	0.0, 101	46.9*	33.4	0.0, 72.6	63.8	47.1	0.0, 91.0	25.3*	0.0	0.0, 38.3
Ruminant meat	33.8	3.7	0.0, 51.6	30.9	0.0	0.0, 47.9	22.2	0.0	0.0, 34.8	48.1*	27.9	0.0, 71.3	30.8	9.2	0.0, 46.6	52.0*	33.8	0.0, 76.0	16.9	0.0	0.0, 16.7
Non-ruminant meat	24.4	0.0	0.0, 33.8	23.5*	0.0	0.0, 31.0	44.6*	17.0	0.0, 69.1	19.9*	0.0	0.0, 28.2	16.1*	0.0	0.0, 19.3	11.8	0.0	0.0, 2.6	8.3	0.0	0.0, 0.0
White meat	37.1	0.0	0.0, 56.3	42.2	0.0	0.0, 64.1	21.1*	0.0	0.0, 28.0	37.0	6.7	0.0, 56.8	39.2	3.0	0.0, 61.2	42.6	7.2	0.0, 63.3	34.9	0.0	0.0, 45.9
Offal meat	2.5	0.0	0.0, 0.0	1.5	0.0	0.0, 0.0	2.1	0.0	0.0, 0.0	1.1	0.0	0.0, 0.0	0.9	0.0	0.0, 0.0	8.2*	0.0	0.0, 0.0	0.9	0.0	0.0, 0.0
Fish and seafood	28.3	0.0	0.0, 40.0	34.3	0.0	0.0, 53.1	7.3*	0.0	0.0, 0.0	21.5*	0.0	0.0, 30.9	27.0*	0.0	0.0, 40.2	43.5*	14.9	0.0, 70.8	34.1	0.0	0.0, 50.2
Fish	24.8	0.0	0.0, 32.7	32.4	0.0	0.0, 49.1	6.8*	0.0	0.0, 0.0	19.4*	0.0	0.0, 27.2	25.7	0.0	0.0, 37.1	28.9	0.0	0.0, 48.4	29.2	0.0	0.0, 42.1
Seafood	3.5	0.0	0.0, 0.0	1.9	0.0	0.0, 0.0	0.4	0.0	0.0, 0.0	2.1	0.0	0.0, 0.0	1.3	0.0	0.0, 0.0	14.6*	0.0	0.0, 0.0	4.9*	0.0	0.0, 0.0
Other protein sources[3]	0.0	0.0	0.0, 0.0	0.1	0.0	0.0, 0.0	0.0	0.0	0.0, 0.0	0.0	0.0	0.0, 0.0	0.0	0.0	0.0, 0.0	0.0	0.0	0.0, 0.0	0.3	0.0	0.0, 0.0
Eggs	20.1	4.9	0.0, 28.3	15.4	0.0	0.0, 19.8	23.6	7.0	0.0, 35.4	12.4	2.5	0.0, 18.2	31.6*	13.8	0.0, 46.1	25.0	12.4	0.0, 36.4	15.5	0.0	0.0, 23.8
Dairy products	245	199	94.0, 340	227*	190	87.4, 320	153	120	56.5, 220	469*	431	279, 603	330*	287	172, 442	207*	181	98.0, 281	164	123	51.4, 236
Milk	136	72.3	0.0, 207	111*	52.4	0.0, 178	76.6	28.2	0.0, 118	392*	351	209, 521	165*	120	15.2, 253	101*	64.7	0.0, 163	67.0	3.4	0.0, 91.1
Yoghurt	56.1	0.0	0.0, 85.2	70.3*	0.0	0.0, 114	28.9	0.0	0.0, 43.0	34.4	0.0	0.0, 48.9	99.2*	53.4	0.0, 150	31.0	0.0	0.0, 43.2	52.3*	0.0	0.0, 81.4
Cream and dessert	18.6	0.0	0.0, 22.5	17.2	0.0	0.0, 20.1	15.1	0.0	0.0, 17.2	19.1	2.3	0.0, 24.5	27.5*	6.3	0.0, 35.2	20.3	0.0	0.0, 26.4	13.1	0.0	0.0, 14.0
Cheese	33.6	25.0	7.2, 48.5	28.1	21.0	1.8, 41.9	32.5	25.5	7.6, 47.5	22.7*	17.0	3.1, 33.7	38.7*	29.8	12.0, 53.3	54.3*	44.8	19.9, 74.6	31.2	23.6	2.1, 47.1
Animal fats	6.4	1.5	0.0, 9.1	6.6*	1.6	0.0, 9.7	8.8*	3.7	0.0, 13.5	4.7	0.9	0.0, 6.0	4.4	0.1	0.0, 5.2	5.5*	1.5	0.0, 7.8	4.0	0.0	0.0, 5.0
**Plant-based foods**																					
Grains	227	221	168, 278	213	208	156, 264	237	231	180, 288	216	211	165, 263	252*	237	178, 307	241*	237	188, 289	227	218	164, 278
Refined grains	193	189	129, 251	191*	185	130, 246	228*	223	170, 280	178*	171	124, 227	110*	105	62.7, 149	232*	230	178, 281	162*	158	102, 219
Breakfast cereals	3.7	0.0	0.0, 0.0	1.5	0.0	0.0, 0.0	0.6	0.0	0.0, 0.0	25.6*	19.7	0.0, 40.2	1.4	0.0	0.0, 0.0	1.1	0.0	0.0, 0.0	8.1*	0.0	0.0, 0.0
Fine bakery wares	44.8	26.2	0.0, 70.6	52.6*	32.8	0.0, 84.3	46.2	28.5	0.0, 74.5	37.3	21.2	0.0, 58.1	40.1	23.8	0.0, 64.1	29.7*	16.4	0.0, 46.2	42.1	23.9	0.0, 67.1
Bread	105	96.8	51.0, 148	103*	95.8	55.4, 142	158*	151	108, 199	77.0	70.2	36.3, 109	45.2*	36.1	2.6, 70.4	109*	104	62.7, 148	75.7	66.1	26.6, 112
Cereals, pasta, rice	39.7	26.2	0.9, 59.1	34.4	23.6	0.0, 52.9	22.7	13.2	0.0, 37.4	38.5	28.0	5.6, 56.3	23.5	15.3	0.0, 36.0	91.8*	84.2	47.8, 124	35.6	23.0	0.0, 55.1
Whole grains	34.4	0.0	0.0, 48.3	22.3*	0.0	0.0, 36.5	9.0	0.0	0.0, 0.0	37.4*	22.7	0.0, 60.8	142*	122	76.3, 184	9.3	0.0	0.0, 4.7	65.1*	40.2	0.0, 97.3
WG breakfast cereals	11.0	0.0	0.0, 0.0	5.8*	0.0	0.0, 0.0	2.3	0.0	0.0, 0.0	11.1*	0.0	0.0, 8.1	50.6*	0.6	0.0, 60.7	2.9	0.0	0.0, 0.0	26.0*	0.0	0.0, 30.1
WG bread	20.9	0.0	0.0, 25.7	15.1*	0.0	0.0, 15.8	5.7	0.0	0.0, 0.0	24.5	0.0	0.0, 41.5	82.2*	73.7	27.2, 122	5.2	0.0	0.0, 0.0	23.2	0.0	0.0, 38.0
WG cereals, pasta, rice	2.5	0.0	0.0, 0.0	1.4	0.0	0.0, 0.0	0.9	0.0	0.0, 0.0	1.8	0.0	0.0, 0.0	9.3*	0.0	0.0, 0.9	1.2	0.0	0.0, 0.0	15.9*	0.0	0.0, 4.9
Starchy roots and tubers	83.1	65.8	0.0, 125	92.4	74.4	1.2, 139	75.1*	60.4	0.0, 114	93.1	79.0	27.0, 136	81.7*	65.6	9.1, 123	63.7	49.0	0.0, 99.3	62.1	36.9	0.0, 98.9
Vegetables	180	144	79.2, 238	197	157	83.9, 262	135*	108	58.6, 179	152*	124	66.9, 199	195	161	93.1, 253	190	163	98.3, 248	246*	205	120, 321
Fruit	160	110	11.8, 234	192	139	21.5, 283	99.6*	59.4	0.0, 152	138	91.1	11.4, 198	177*	136	48.5, 253	142	107	21.1, 210	202	151	53.4, 297
Legumes	9.4	0.0	0.0, 6.2	7.8*	0.0	0.0, 2.5	9.5	0.0	0.0, 4.2	12.7*	0.0	0.0, 12.5	10.8	0.0	0.0, 9.0	9.8	0.0	0.0, 10.0	17.5*	0.0	0.0, 20.9
Nuts and seeds	4.1	0.0	0.0, 0.0	3.5	0.0	0.0, 0.0	3.7	0.0	0.0, 0.0	2.7	0.0	0.0, 0.0	8.5*	0.0	0.0, 8.4	2.5	0.0	0.0, 0.0	17.1*	2.1	0.0, 22.3
Vegetable oils and fats	16.9	13.7	5.4, 25.1	16.0	12.8	4.7, 23.7	15.0	11.6	4.1, 22.5	14.8	12.2	5.3, 21.4	17.6*	14.5	6.7, 25.5	22.8*	21.4	10.0, 33.8	15.6	12.6	5.8, 21.6
Meat and dairy imitates	5.1	0.0	0.0, 0.0	2.3	0.0	0.0, 0.0	0.6	0.0	0.0, 0.0	1.4	0.0	0.0, 0.0	3.0	0.0	0.0, 0.0	1.1	0.0	0.0, 0.0	211*	175	72.5, 299

Meat imitates	0.7	0.0	0.0, 0.0	0.2	0.0	0.0, 0.0	0.0	0.0	0.0, 0.0	0.3	0.0	0.0, 0.0	0.4	0.0	0.0, 0.0	0.2	0.0	0.0, 0.0	32.3*	0.0	0.0, 57.9
Dairy imitates	4.4	0.0	0.0, 0.0	2.2	0.0	0.0, 0.0	0.5	0.0	0.0, 0.0	1.1	0.0	0.0, 0.0	2.5	0.0	0.0, 0.0	0.9	0.0	0.0, 0.0	179*	154	0.0, 286
**Mixed foods**																					
Sugar and confectionary	29.7	19.4	4.8, 40.7	33.3*	21.3	5.0, 45.1	27.1	17.6	3.9, 38.0	28.7	20.5	5.5, 40.2	28.3	18.4	3.5, 40.2	25.4	17.7	6.4, 33.9	26.0	14.2	2.2, 36.5
Composite dishes	30.9	0.0	0.0, 3.2	39.5*	0.0	0.0, 11.2	28.3	0.0	0.0, 9.5	34.3	0.0	0.0, 12.0	23.3	0.0	0.0, 0.0	14.4*	0.0	0.0, 0.0	27.3	0.0	0.0, 0.0
Miscellaneous	27.9	14.4	4.8, 35.5	30.8	15.8	4.8, 39.6	23.5	14.6	5.6, 30.8	28.5	14.6	5.0, 38.5	28.9	15.9	5.9, 38.3	23.3	9.6	3.3, 25.8	32.9	17.2	6.0, 44.6
**Beverages**																					
Hot beverages	519	367	114, 730	634*	473	169, 885	422*	309	89.5, 599	465*	334	69.9, 696	577	476	225, 780	302*	166	62.2, 400	553	365	108, 766
Alcoholic beverages	158	2.3	0.0, 215	153	0.0	0.0, 203	220*	51.3	0.0, 320	138	1.9	0.0, 176	114	0.0	0.0, 149	149	37.9	0.0, 216	96.6	0.0	0.0, 131
Sweetened beverages	205	94.9	0.0, 288	235*	109	0.0, 331	213	103	0.0, 306	204	118	0.0, 292	162	82.8	0.0, 239	153	59.5	0.0, 213	150	78.5	0.0, 226
Drinking water	1127	893	445, 1550	1328	1076	514, 1857	922	764	385, 1295	973	764	359, 1358	1097*	885	449, 1513	936	753	428, 1265	1298	1080	575, 1777

IQR interquartile range, *Mdn* median, *WG* wholegrain.

[1] Food consumption was standardized to a reference energy intake of 2000 kcal.

[2] Meat does not include all consumed meat products, as a small part of meat products is consumed in the form of composite dishes.

[3] Other protein sources include amphibians, reptiles, snails and insects.

* Pairwise t-test comparison, with Bonferroni correction, for consumption that was significantly different from all other dietary protein patterns.

Supplemental Table 6 Associations[1] between socio-demographic and anthropometric characteristics and the six dietary protein patterns, obtained from the EFSA Comprehensive European Food Consumption Database [1].

	Fast-food		Milk-rich		Health-conscious		Traditional		Plant-forward	
	OR	95% CI	OR	95% CI	OR	95% CI	OR	95% CI	OR	95% CI
**Sex**										
Females (reference)	1.00		1.00		1.00		1.00		1.00	
Males	4.96	4.67, 5.27	2.50	2.32, 2.69	2.14	1.99, 2.29	2.99	2.79, 3.19	1.47	1.24, 1.74
**Age category**										
18-34 years (reference)	1.00		1.00		1.00		1.00		1.00	
35-49 years	1.03	0.96, 1.11	0.72	0.66, 0.78	1.04	0.96, 1.13	0.90	0.83, 0.98	0.89	0.73, 1.08
50-64 years	0.95	0.88, 1.02	0.53	0.48, 0.58	1.19	1.09, 1.29	0.74	0.68, 0.81	0.73	0.59, 0.91
**Weight status[2]**										
Underweight	1.01	0.78, 1.30	1.16	0.89, 1.50	0.90	0.69, 1.17	1.26	1.00, 1.58	1.47	0.92, 2.32
Normal weight (reference)	1.00		1.00		1.00		1.00		1.00	
Overweight	1.00	0.93, 1.07	0.85	0.78, 0.92	0.72	0.67, 0.78	0.82	0.76, 0.88	0.53	0.44, 0.64
Obese	1.11	1.02, 1.22	1.03	0.92, 1.14	0.84	0.76, 0.92	0.75	0.68, 0.83	0.50	0.38, 0.65
**Region[3]**										
Western Europe (reference)	1.00		1.00		1.00		1.00		1.00	
Eastern Europe	1.74	1.62, 1.86	0.51	0.45, 0.58	1.21	1.10, 1.32	2.24	2.05, 2.46	0.28	0.20, 0.40
Northern Europe	0.51	0.47, 0.56	1.60	1.46, 1.74	2.41	2.23, 2.61	1.36	1.22, 1.51	0.83	0.66, 1.05
Southern Europe	0.34	0.31, 0.39	1.05	0.94, 1.16	0.27	0.23, 0.32	5.96	5.49, 6.48	1.50	1.23, 1.83

CI confidence interval, *OR* odds ratio.

[1] Multinomial logistic regression models were adjusted for all variables presented in this table. The *Common* protein pattern was used as a reference. The French population (n = 1726) was not included in the analysis due to missing values for age.

[2] Based on BMI cut-off values of the World Health Organization [7]: underweight (<18.5), normal weight (18.5-24.9), overweight (25.0-29.9) and obese (≥30.0).

[3] Based on geographical classification of EuroVoc [8]: Northern Europe (Denmark, Estonia, Finland, Latvia and Sweden), Southern Europe (Cyprus, Greece, Italy, Portugal and Spain), Eastern Europe (Bosnia and Herzegovina, Croatia, Czechia, Hungary, Montenegro, Romania, Serbia and Slovenia) and Western Europe (Austria, Belgium, France, Germany, Ireland, Netherlands and United Kingdom).

Supplemental Table 7 Protein intake of the total study population and the six dietary protein patterns, obtained from the EFSA Comprehensive European Food Consumption Database [1].

	**Total**			**Common**			**Fast-food**			**Milk-rich**			**Health-conscious**			**Traditional**			**Plant-forward**		
	Mean	SD	%[1]	Mean	SD	%[1]	Mean	SD	%[1]	Mean	SD	%[1]	Mean	SD	%[1]	Mean	SD	%[1]	Mean	SD	%[1]
**Total protein (E%)**	16.4	4.6		16.0	4.9		15.9	3.8		17.0	4.3		17.0	4.5		17.4	4.7		16.0	5.3	
**Total protein (g/day)**	79.2	30.3		63.8	23.4		93.4	28.2		85.9	29.1		86.7	28.4		93.6	32.0		80.0	33.2	
Animal protein	52.7	26.3	66.5%	42.3	21.6	66.3%	60.2	25.0	64.4%	59.5	25.7	69.2%	57.8	25.6	66.7%	64.8	29.3	69.2%	41.8	31.1	52.2%
Plant protein	26.5	10.6	33.5%	21.5	7.6	33.7%	33.2	11.2	35.6%	26.5	9.4	30.8%	28.9	10.4	33.3%	28.8	10.0	30.8%	38.2	13.9	47.8%
**Food group protein (g/day)**																					
Animal-based foods																					
Meat[2]	27.7	20.9	35.0%	21.4	16.1	33.5%	39.5	22.9	42.3%	29.8	21.0	34.7%	26.0	19.3	30.0%	31.3	23.3	33.4%	16.6	21.4	20.8%
Processed meat	7.7	9.0	9.7%	4.8	5.3	7.5%	16.9	12.3	18.1%	6.7	7.5	7.8%	6.9	7.7	7.9%	5.5	5.7	5.8%	3.5	6.2	4.3%
Red meat	11.9	14.3	15.0%	9.0	10.8	14.1%	16.4	17.8	17.6%	14.7	15.9	17.1%	10.1	12.1	11.7%	14.4	16.4	15.4%	5.2	9.6	6.5%
Ruminant meat	6.8	10.8	8.6%	5.1	8.2	8.0%	5.5	9.3	5.9%	10.3	13.7	12.0%	6.5	9.7	7.5%	11.7	15.1	12.5%	3.4	7.3	4.3%
Non-ruminant meat	5.1	9.9	6.4%	3.9	7.4	6.1%	11.0	15.5	11.7%	4.4	8.3	5.1%	3.6	7.6	4.2%	2.7	6.4	2.9%	1.8	5.8	2.2%
White meat	7.8	12.9	9.8%	7.4	12.0	11.6%	5.8	11.3	6.2%	8.2	12.6	9.6%	8.9	13.4	10.2%	10.3	16.0	11.0%	7.8	16.0	9.8%
Offal meat	0.4	2.3	0.5%	0.2	1.2	0.3%	0.5	2.4	0.5%	0.2	1.3	0.2%	0.2	1.3	0.2%	1.2	4.5	1.2%	0.1	1.1	0.2%
Fish and seafood	6.2	12.7	7.8%	6.6	13.0	10.4%	2.1	6.6	2.3%	5.0	9.6	5.8%	6.0	10.5	6.9%	11.2	17.7	12.0%	8.1	15.6	10.2%
Fish	5.5	11.9	7.0%	6.4	12.9	10.0%	2.0	6.5	2.2%	4.6	9.4	5.3%	5.8	10.4	6.6%	8.1	15.3	8.6%	7.1	14.8	8.9%
Seafood	0.7	3.7	0.9%	0.3	1.6	0.5%	0.1	1.1	0.1%	0.4	2.0	0.5%	0.2	1.4	0.3%	3.1	8.4	3.3%	1.0	4.4	1.2%
Other protein sources[3]	0.0	0.3	0.0%	0.0	0.5	0.0%	0.0	0.0	0.0%	0.0	0.0	0.0%	0.0	0.0	0.0%	0.0	0.2	0.0%	0.1	1.1	0.1%
Eggs	2.3	3.6	2.9%	1.4	2.3	2.2%	3.1	4.4	3.4%	1.5	2.4	1.8%	3.6	5.0	4.2%	3.2	4.1	3.4%	1.8	3.2	2.3%
Dairy products	13.9	10.4	17.6%	10.3	7.4	16.2%	13.0	9.9	13.9%	20.5	11.6	23.8%	18.6	11.9	21.5%	17.5	11.2	18.7%	11.7	10.0	14.6%
Milk	4.6	6.1	5.7%	3.1	4.2	4.8%	3.2	4.6	3.4%	13.4	9.2	15.6%	5.9	6.4	6.8%	3.7	4.6	4.0%	2.4	4.1	3.0%
Yoghurt	1.9	3.5	2.4%	2.1	3.2	3.2%	1.3	2.6	1.4%	1.2	2.6	1.4%	3.8	5.6	4.4%	1.2	2.5	1.3%	2.0	4.1	2.5%
Cream and dessert	0.5	1.2	0.7%	0.4	0.9	0.6%	0.5	1.1	0.5%	0.6	1.2	0.7%	0.9	1.8	1.0%	0.6	1.4	0.7%	0.4	1.0	0.5%
Cheese	6.9	7.5	8.8%	4.8	5.1	7.5%	7.9	8.1	8.5%	5.3	5.6	6.1%	8.1	7.8	9.3%	11.9	9.9	12.7%	6.8	7.6	8.5%
Animal fats	0.0	0.1	0.1%	0.0	0.1	0.1%	0.1	0.1	0.1%	0.0	0.1	0.0%	0.0	0.1	0.0%	0.0	0.1	0.0%	0.0	0.1	0.0%
Plant-based foods																					
Grains	18.1	9.1	22.9%	14.1	6.7	22.0%	24.4	10.1	26.2%	17.8	8.1	20.7%	19.4	8.8	22.4%	20.5	8.7	21.9%	18.3	9.7	22.8%
Refined grains	15.6	9.3	19.7%	12.6	6.7	19.8%	23.6	10.2	25.3%	14.6	7.7	17.0%	9.1	6.4	10.5%	19.7	8.8	21.0%	13.4	9.2	16.7%
Breakfast cereals	0.3	1.1	0.4%	0.1	0.4	0.2%	0.1	0.4	0.1%	2.2	2.5	2.5%	0.1	0.5	0.2%	0.1	0.5	0.1%	0.8	2.2	0.9%
Fine bakery wares	2.8	3.9	3.5%	2.8	3.9	4.4%	3.5	4.8	3.8%	2.5	3.5	2.9%	2.7	3.6	3.1%	2.2	2.9	2.3%	2.8	4.1	3.5%
Bread	10.3	8.0	13.0%	8.3	5.5	12.9%	18.4	9.0	19.7%	7.9	6.2	9.1%	4.9	5.1	5.6%	11.8	7.6	12.6%	8.0	7.4	10.0%
Cereals, pasta, rice	2.2	2.8	2.7%	1.4	1.7	2.2%	1.7	2.3	1.8%	2.1	2.3	2.4%	1.4	1.8	1.6%	5.6	4.2	6.0%	1.8	2.4	2.3%
Whole grains	2.5	4.5	3.2%	1.5	2.4	2.3%	0.8	2.0	0.9%	3.2	4.1	3.7%	10.3	6.5	11.9%	0.9	2.0	0.9%	4.9	6.2	6.1%
WG breakfast cereals	0.7	2.0	0.8%	0.4	1.2	0.6%	0.2	1.0	0.2%	0.9	2.1	1.0%	2.6	4.1	3.0%	0.3	1.1	0.3%	1.8	3.5	2.3%
WG bread	1.7	3.7	2.2%	1.0	2.1	1.6%	0.5	1.7	0.6%	2.2	3.3	2.5%	7.2	6.4	8.3%	0.5	1.6	0.5%	2.0	3.4	2.5%
WG cereals, pasta, rice	0.2	0.9	0.2%	0.1	0.4	0.1%	0.1	0.5	0.1%	0.1	0.7	0.2%	0.6	1.5	0.6%	0.1	0.5	0.1%	1.1	3.7	1.4%
Starchy roots and tubers	1.6	1.7	2.0%	1.5	1.5	2.3%	1.8	1.9	1.9%	1.9	1.8	2.2%	1.7	1.8	2.0%	1.4	1.6	1.5%	1.3	1.7	1.6%
Vegetables	2.1	1.7	2.6%	1.9	1.6	3.0%	1.9	1.6	2.0%	1.9	1.5	2.2%	2.4	1.9	2.8%	2.5	1.9	2.7%	3.3	2.5	4.1%
Fruit	0.9	1.1	1.2%	0.9	1.0	1.5%	0.7	0.9	0.7%	0.9	1.1	1.0%	1.2	1.2	1.4%	1.0	1.0	1.0%	1.4	1.6	1.7%

Legumes	0.6	1.6	0.8%	0.4	1.2	0.6%	0.7	1.8	0.7%	0.9	1.9	1.0%	0.7	1.7	0.8%	0.7	1.7	0.8%	1.4	3.0	1.7%
Nuts and seeds	0.9	2.9	1.1%	0.6	2.0	1.0%	1.0	3.2	1.1%	0.6	1.9	0.7%	1.9	4.2	2.2%	0.6	2.2	0.6%	3.8	7.2	4.7%
Vegetable oils and fats	0.0	0.0	0.0%	0.0	0.0	0.0%	0.0	0.0	0.0%	0.0	0.0	0.0%	0.0	0.1	0.0%	0.0	0.0	0.0%	0.0	0.0	0.0%
Meat and dairy imitates	0.2	1.3	0.2%	0.1	0.4	0.1%	0.0	0.3	0.0%	0.1	0.4	0.1%	0.1	0.7	0.1%	0.1	0.4	0.1%	7.8	6.3	9.7%
Meat imitates	0.1	1.0	0.1%	0.0	0.2	0.0%	0.0	0.1	0.0%	0.0	0.3	0.0%	0.1	0.5	0.1%	0.0	0.3	0.0%	4.0	6.2	5.0%
Dairy imitates	0.1	0.9	0.1%	0.0	0.4	0.1%	0.0	0.2	0.0%	0.0	0.3	0.0%	0.1	0.4	0.1%	0.0	0.3	0.0%	3.8	4.9	4.7%
Mixed foods																					
Sugar and confectionary	0.6	1.2	0.8%	0.6	1.2	0.9%	0.7	1.3	0.7%	0.7	1.3	0.8%	0.7	1.3	0.8%	0.5	1.2	0.5%	0.6	1.1	0.8%
Composite dishes	1.4	4.2	1.7%	1.4	4.3	2.2%	1.3	4.1	1.4%	1.9	5.3	2.2%	1.3	4.5	1.5%	0.9	3.3	1.0%	1.1	3.5	1.3%
Miscellaneous	0.9	5.0	1.2%	0.9	6.4	1.4%	0.7	1.2	0.7%	0.9	3.1	1.1%	1.2	5.2	1.4%	0.9	3.8	1.0%	1.4	8.4	1.8%
Beverages																					
Hot beverages	0.9	1.6	1.1%	0.9	1.7	1.5%	1.0	1.8	1.1%	0.8	1.4	0.9%	0.9	1.5	1.1%	0.6	1.2	0.6%	0.6	1.3	0.8%
Alcoholic beverages	0.5	1.3	0.7%	0.4	1.1	0.6%	1.0	1.9	1.0%	0.5	1.2	0.6%	0.4	1.1	0.5%	0.5	1.2	0.5%	0.3	0.9	0.4%
Sweetened beverages	0.3	0.7	0.4%	0.3	0.7	0.5%	0.3	0.8	0.4%	0.3	0.7	0.4%	0.4	0.7	0.4%	0.3	0.6	0.3%	0.4	0.8	0.5%
Drinking water	0.0	0.0	0.0%	0.0	0.0	0.0%	0.0	0.0	0.0%	0.0	0.0	0.0%	0.0	0.0	0.0%	0.0	0.0	0.0%	0.0	0.0	0.0%

SD standard deviation, *WG* wholegrain.

[1] Percentage of total protein.

[2] Meat does not include all consumed meat products, as a small part of meat products is consumed in the form of composite dishes.

[3] Other protein sources include amphibians, reptiles, snails and insects.

Supplemental Table 8 Energy-standardized[1] nutrient intakes, nutritional adequacies and Nutrient Rich Diet scores of the total study population and the six dietary protein patterns, obtained from the EFSA Comprehensive European Food Consumption Database [1].

	Type of DRV[2]	Total			Common			Fast-food			Milk-rich			Health-conscious			Traditional			Plant-forward		
		Mean	SD	%DRV	Mean	SD	%DRV	Mean	SD	%DRV	Mean	SD	%DRV	Mean	SD	%DRV	Mean	SD	%DRV	Mean	SD	%DRV
**Other nutrients**																						
Energy (kcal/day)		1978	671		1643*	522		2416*	682		2066	644		2092	620		2191*	620		2054	672	
EPA+DHA (mg/day)	AI	330	715	132%	401	843	160%	126*	301	51%	236*	499	95%	327	631	131%	456	803	182%	407	886	163%
Vit B3 (mg NE/MJ)	AR	2.50	1.10	193%	2.53	1.17	194%	2.43	0.93	187%	2.66*	1.12	205%	2.45	1.01	188%	2.49	1.09	192%	2.31	1.22	178%
	PRI			156%			158%			152%			166%			153%			156%			144%
Vit B6 (mg/day)	AR	1.84	0.77	132%	1.83	0.80	134%	1.72	0.68	120%	2.09*	0.81	149%	1.93*	0.72	138%	1.81	0.78	127%	1.79	0.80	131%
	PRI			112%			112%			103%			126%			117%			109%			109%
Iodine (µg/day)	AI	228	131	152%	223*	150	148%	228	99.3	152%	235	109	157%	233	100	155%	238	147	159%	185*	102	124%
Magnesium (mg/day)	AI	325	87.3	101%	319*	85.7	101%	303*	68.1	91%	357*	90.3	110%	372*	85.3	115%	296*	76.9	90%	441*	124	139%
Phosphorus (mg/day)	AI	1517	395	276%	1416*	374	258%	1483	327	270%	1730*	419	315%	1702*	415	309%	1553	389	282%	1519	399	276%
Selenium (µg/day)	AI	54.9	32.7	78%	53.4	36.9	76%	52.1	19.6	74%	53.5	25.9	76%	57.9	33.6	83%	61.1*	35.1	87%	54.5	41.7	78%
**Qualifying nutrients**																						
Protein (g/kg BW)	AR	1.23	0.40	187%	1.20*	0.41	181%	1.18*	0.34	179%	1.29	0.39	196%	1.26	0.39	191%	1.35*	0.42	204%	1.26	0.46	190%
	PRI			149%			144%			142%			156%			152%			162%			151%
Fiber (g/day)	AI	21.8	7.17	87%	21.5*	7.21	86%	21.0*	6.17	84%	22.5*	8.08	90%	24.7*	7.48	99%	20.1*	6.00	80%	27.8*	9.17	111%
MUFA (E%)	RI	12.9	3.90	129%	12.6	3.94	126%	13.5	3.26	135%	11.6*	3.26	116%	12.5	3.66	125%	14.4*	4.51	144%	13.5	4.34	135%
Vit A (µg RE/day)	AR	1467	2696	279%	1348	2009	263%	1454	2411	267%	1297	1731	244%	1424	1579	271%	1989*	5038	373%	1289	1248	250%
	PRI			211%			199%			202%			185%			205%			282%			189%
Vit B12 (µg/day)	AI	6.15	10.3	154%	5.39	7.35	135%	5.20	8.36	130%	6.51*	6.77	163%	5.84	5.38	146%	9.71*	20.0	243%	5.12	4.30	128%
Vit B1 (mg/1000 kcal)	AR	0.54	0.21	180%	0.52*	0.21	173%	0.60	0.24	199%	0.61	0.21	202%	0.55	0.19	185%	0.46*	0.15	154%	0.55	0.22	182%
	PRI			135%			130%			150%			151%			138%			115%			136%
Vit B2 (mg/day)	AR	1.47	0.60	113%	1.33*	0.52	102%	1.38*	0.48	106%	1.96*	0.64	151%	1.64	0.57	126%	1.52	0.73	117%	1.57	0.62	121%
	PRI			92%			83%			86%			123%			102%			95%			98%
Vit C (mg/day)	AR	105	79.4	125%	113	87.6	137%	90.6*	68.4	104%	100	69.4	118%	113	78.4	134%	97.3	69.1	114%	125*	92.7	150%
	PRI			104%			114%			86%			98%			112%			94%			125%
Vit D (µg/day)	AI	3.21	2.85	21%	3.22*	3.15	21%	2.94	1.78	20%	3.02	2.18	20%	3.87*	3.22	26%	3.01	2.96	20%	4.35*	3.72	29%
Vit E (mg/day)	AI	12.3	5.84	103%	12.0*	5.90	104%	12.4	5.95	100%	11.0*	4.53	91%	13.5*	5.68	114%	12.6	5.91	104%	14.6*	7.64	125%
Folate (µg DFE/day)	AR	274	114	110%	262*	115	105%	254*	90.5	102%	311*	129	124%	291	104	116%	293	122	117%	339*	132	136%
	PRI			83%			79%			77%			94%			88%			89%			103%
Calcium (mg/day)	AR	888	370	116%	844*	342	111%	748*	294	97%	1136*	412	147%	1070	405	141%	864*	337	112%	1069	387	141%
	PRI			93%			88%			78%			118%			112%			89%			112%
Iron (mg/day)	AR	10.9	3.56	177%	10.4*	3.51	167%	10.9*	3.03	179%	11.3	3.56	182%	12.0*	3.40	195%	11.1	4.11	181%	12.5*	3.55	200%
	PRI			93%			87%			96%			95%			103%			96%			103%
Potassium (mg/day)	AI	3483	945	100%	3532	980	101%	3232	804	92%	3835*	959	110%	3756*	931	107%	3209	849	92%	3590	944	103%
Zinc (mg/day)	AR	10.5	3.49	154%	9.56*	3.27	145%	11.0	3.01	155%	11.6	3.60	168%	11.4	3.27	168%	11.2	4.06	161%	10.3*	3.27	154%
	PRI			126%			119%			125%			136%			136%			130%			126%

**Disqualifying nutrients**																						
Sugar (E%)	UL	19.5	8.11	195%	21.4	8.52	214%	16.6*	7.59	166%	21.4	7.50	214%	19.9	7.10	199%	16.2*	6.58	162%	20.6	7.03	206%
SFA (E%)	UL	13.2	4.02	132%	12.8	4.02	128%	14.3*	3.97	143%	12.9	3.72	129%	13.3	3.92	133%	13.4	4.03	134%	11.6*	4.01	115%
Sodium (mg/day)	UL	3227	1793	161%	3042	1841	152%	3874*	1800	194%	2985	1460	149%	3058	1698	153%	3252*	1762	163%	2747*	1312	137%
**NRD scores**																						
NRD15	AR/AI/RI	1302	95.4		1289*	104		1280*	90.9		1329*	72.3		1348	75.8		1306*	87.2		1342	83.6	
	PRI/AI/RI	1229	123		1206	131		1207	115		1274	98.1		1290	103		1234*	116		1284	111	
NRDX.3	UL	489	102		494	107		503*	96.8		492	92.0		485*	97.5		458	101		459	84.6	
NRD15.3	AR/AI/RI	813	142		795*	153		777*	130		837*	120		863*	124		847*	128		883*	111	
	PRI/AI/RI	740	161		712*	173		704*	146		782	137		806*	141		776	145		825*	131	

AI adequate intake, *AR* average requirement, *BW* body weight, *DFE* dietary folate equivalents, *DHA* docosahexaenoic acid, *DRV* dietary reference value, *E%* energy percentage, *EPA* eicosapentaenoic acid, *MUFA* monounsaturated fatty acids, *NRD* Nutrient Rich Diet, *PRI* population reference intake, *RE* retinol equivalents, *RI* reference intake, *SD* standard deviation, *SFA* saturated fatty acids, *UL* upper limit, *Vit* vitamin.

[1] Nutrient intakes were standardized to a reference energy intake of 2000 kcal for females and 2500 kcal for males.

[2] Type of DRV used to calculate nutritional adequacy percentages and NRD scores. Information on the applied DRVs is provided in Supplemental Table 2.

* Pairwise t-test comparison, with Bonferroni correction, for intake that was significantly different from all other dietary protein patterns.

Supplemental Table 9 Diet-related greenhouse gas emissions of the total study population and the six dietary protein patterns, obtained from the EFSA Comprehensive European Food Consumption Database [1].

	Total			Common			Fast-food			Milk-rich			Health-conscious			Traditional			Plant-forward		
	Mean	SD	%[1]	Mean	SD	%[1]	Mean	SD	%[1]	Mean	SD	%[1]	Mean	SD	%[1]	Mean	SD	%[1]	Mean	SD	%[1]
**Total GHGE**																					
Absolute (kg CO_2-eq/day)	5.71	2.36		4.81	1.86		6.71	2.31		6.11	2.52		5.84	2.15		6.67	2.75		4.70	2.07	
Standardized (kg CO_2-eq/2000 kcal)	5.89	1.87		5.97	1.83		5.65	1.52		6.01	2.03		5.68	1.67		6.21*	2.27		4.69*	1.76	
**Food group GHGE (kg CO_2-eq/day)**																					
Animal-based foods																					
Meat[2]	2.17	1.84	38.1%	1.59	1.34	33.2%	2.93	1.80	43.7%	2.53	2.13	41.4%	2.00	1.65	34.3%	2.83	2.40	42.4%	1.11	1.43	23.7%
Processed meat	0.55	0.69	9.7%	0.34	0.40	7.0%	1.27	0.98	18.9%	0.47	0.55	7.7%	0.48	0.58	8.3%	0.38	0.44	5.7%	0.23	0.45	4.8%
Red meat	1.28	1.65	22.5%	0.96	1.26	20.0%	1.41	1.57	21.0%	1.73	2.02	28.3%	1.18	1.48	20.2%	1.91	2.28	28.7%	0.59	1.10	12.5%
Ruminant meat	1.00	1.58	17.5%	0.74	1.20	15.4%	0.79	1.33	11.7%	1.48	1.96	24.3%	0.97	1.43	16.7%	1.75	2.26	26.3%	0.49	1.03	10.3%
Non-ruminant meat	0.29	0.57	5.0%	0.22	0.42	4.6%	0.62	0.88	9.2%	0.25	0.48	4.1%	0.21	0.43	3.5%	0.16	0.38	2.4%	0.10	0.33	2.1%
White meat	0.29	0.48	5.1%	0.28	0.45	5.8%	0.22	0.42	3.2%	0.31	0.47	5.0%	0.33	0.50	5.6%	0.39	0.60	5.8%	0.29	0.59	6.2%
Offal meat	0.04	0.25	0.7%	0.02	0.13	0.4%	0.04	0.23	0.6%	0.02	0.16	0.3%	0.01	0.11	0.2%	0.14	0.52	2.1%	0.01	0.06	0.2%
Fish and seafood	0.26	0.59	4.6%	0.26	0.52	5.5%	0.09	0.30	1.4%	0.19	0.39	3.1%	0.25	0.48	4.2%	0.55	1.02	8.2%	0.31	0.62	6.5%
Fish	0.20	0.45	3.6%	0.24	0.49	4.9%	0.08	0.28	1.2%	0.16	0.33	2.5%	0.22	0.45	3.8%	0.29	0.56	4.3%	0.22	0.45	4.7%
Seafood	0.06	0.36	1.1%	0.03	0.18	0.6%	0.01	0.10	0.1%	0.04	0.19	0.6%	0.03	0.15	0.4%	0.26	0.82	3.9%	0.08	0.41	1.8%
Other protein sources[3]	0.00	0.03	0.0%	0.00	0.05	0.0%	0.00	0.00	0.0%	0.00	0.00	0.0%	0.00	0.00	0.0%	0.00	0.02	0.0%	0.00	0.08	0.1%
Eggs	0.04	0.07	0.8%	0.03	0.04	0.5%	0.06	0.09	0.9%	0.03	0.05	0.5%	0.07	0.10	1.2%	0.06	0.08	0.8%	0.03	0.06	0.7%
Dairy products	0.89	0.65	15.7%	0.67	0.46	13.8%	0.87	0.64	13.0%	1.16	0.64	18.9%	1.18	0.73	20.2%	1.18	0.78	17.6%	0.74	0.63	15.8%
Milk	0.21	0.27	3.8%	0.15	0.17	3.0%	0.16	0.21	2.3%	0.63	0.41	10.2%	0.28	0.29	4.7%	0.17	0.20	2.6%	0.11	0.19	2.4%
Yoghurt	0.09	0.15	1.5%	0.09	0.14	2.0%	0.06	0.11	0.9%	0.06	0.11	0.9%	0.17	0.22	2.8%	0.06	0.11	0.9%	0.09	0.15	1.8%
Cream and dessert	0.15	0.28	2.6%	0.12	0.23	2.5%	0.15	0.28	2.2%	0.16	0.27	2.6%	0.21	0.36	3.7%	0.18	0.34	2.7%	0.10	0.22	2.2%
Cheese	0.44	0.48	7.7%	0.31	0.32	6.3%	0.51	0.50	7.6%	0.31	0.34	5.1%	0.52	0.51	8.9%	0.76	0.66	11.4%	0.44	0.49	9.4%
Animal fats	0.21	0.37	3.6%	0.18	0.31	3.7%	0.34	0.53	5.1%	0.16	0.30	2.6%	0.15	0.30	2.6%	0.20	0.34	3.0%	0.14	0.30	3.0%
Plant-based foods																					
Grains	0.56	0.64	9.8%	0.52	0.64	10.9%	0.72	0.77	10.7%	0.49	0.53	8.0%	0.56	0.59	9.7%	0.50	0.50	7.4%	0.54	0.62	11.4%
Refined grains	0.53	0.64	9.3%	0.51	0.64	10.5%	0.71	0.77	10.6%	0.46	0.54	7.5%	0.42	0.58	7.3%	0.49	0.50	7.3%	0.48	0.63	10.2%
Breakfast cereals	0.00	0.01	0.0%	0.00	0.00	0.0%	0.00	0.00	0.0%	0.02	0.02	0.3%	0.00	0.00	0.0%	0.00	0.00	0.0%	0.01	0.01	0.1%
Fine bakery wares	0.40	0.63	7.0%	0.41	0.64	8.5%	0.51	0.77	7.6%	0.33	0.53	5.5%	0.36	0.58	6.2%	0.30	0.49	4.5%	0.37	0.62	7.9%
Bread	0.10	0.08	1.8%	0.08	0.05	1.6%	0.18	0.09	2.7%	0.08	0.06	1.3%	0.05	0.05	0.8%	0.12	0.07	1.7%	0.08	0.07	1.6%
Cereals, pasta, rice	0.03	0.04	0.5%	0.02	0.02	0.4%	0.02	0.03	0.3%	0.03	0.03	0.5%	0.02	0.02	0.3%	0.07	0.05	1.1%	0.03	0.03	0.6%
Whole grains	0.03	0.07	0.5%	0.02	0.03	0.3%	0.01	0.02	0.1%	0.03	0.05	0.6%	0.14	0.12	2.4%	0.01	0.02	0.1%	0.06	0.10	1.2%
WG breakfast cereals	0.01	0.05	0.2%	0.00	0.01	0.1%	0.00	0.01	0.0%	0.01	0.03	0.1%	0.06	0.12	1.0%	0.00	0.01	0.0%	0.03	0.08	0.5%
WG bread	0.02	0.04	0.3%	0.01	0.02	0.2%	0.01	0.02	0.1%	0.02	0.04	0.4%	0.08	0.07	1.3%	0.01	0.02	0.1%	0.02	0.04	0.4%
WG cereals, pasta, rice	0.00	0.01	0.0%	0.00	0.00	0.0%	0.00	0.00	0.0%	0.00	0.01	0.0%	0.01	0.02	0.1%	0.00	0.00	0.0%	0.01	0.03	0.2%
Starchy roots and tubers	0.05	0.05	0.8%	0.04	0.05	0.9%	0.05	0.05	0.8%	0.06	0.06	0.9%	0.05	0.06	0.8%	0.04	0.05	0.6%	0.04	0.05	0.8%
Vegetables	0.27	0.28	4.8%	0.26	0.28	5.3%	0.28	0.29	4.2%	0.23	0.24	3.8%	0.31	0.30	5.2%	0.29	0.27	4.4%	0.35	0.32	7.4%
Fruit	0.10	0.11	1.7%	0.10	0.11	2.0%	0.08	0.10	1.1%	0.09	0.11	1.4%	0.12	0.12	2.0%	0.10	0.10	1.4%	0.13	0.13	2.8%

Legumes	0.01	0.02	0.1%	0.01	0.02	0.1%	0.01	0.03	0.1%	0.01	0.03	0.2%	0.01	0.03	0.2%	0.01	0.03	0.1%	0.02	0.04	0.4%
Nuts and seeds	0.01	0.03	0.2%	0.01	0.02	0.1%	0.01	0.03	0.1%	0.01	0.02	0.1%	0.02	0.05	0.3%	0.01	0.02	0.1%	0.04	0.09	0.9%
Vegetable oils and fats	0.06	0.06	1.1%	0.05	0.05	1.0%	0.07	0.07	1.0%	0.06	0.06	1.0%	0.07	0.07	1.2%	0.09	0.07	1.4%	0.06	0.06	1.3%
Meat and dairy imitates	0.00	0.03	0.1%	0.00	0.01	0.0%	0.00	0.01	0.0%	0.00	0.01	0.0%	0.00	0.01	0.1%	0.00	0.01	0.0%	0.17	0.12	3.6%
Meat imitates	0.00	0.02	0.0%	0.00	0.00	0.0%	0.00	0.00	0.0%	0.00	0.01	0.0%	0.00	0.01	0.0%	0.00	0.01	0.0%	0.07	0.11	1.5%
Dairy imitates	0.00	0.02	0.0%	0.00	0.01	0.0%	0.00	0.01	0.0%	0.00	0.01	0.0%	0.00	0.01	0.0%	0.00	0.01	0.0%	0.10	0.11	2.1%
Mixed foods																					
Sugar and confectionary	0.08	0.12	1.3%	0.07	0.11	1.5%	0.09	0.13	1.3%	0.08	0.12	1.3%	0.08	0.12	1.4%	0.06	0.11	0.9%	0.07	0.11	1.6%
Composite dishes	0.18	0.55	3.2%	0.21	0.57	4.3%	0.19	0.55	2.8%	0.23	0.68	3.8%	0.16	0.54	2.7%	0.10	0.39	1.4%	0.15	0.47	3.1%
Miscellaneous	0.07	0.16	1.1%	0.06	0.14	1.2%	0.06	0.11	0.9%	0.08	0.16	1.2%	0.09	0.25	1.6%	0.06	0.17	0.9%	0.07	0.16	1.5%
Beverages																					
Hot beverages	0.33	0.35	5.8%	0.35	0.36	7.3%	0.34	0.36	5.1%	0.32	0.33	5.3%	0.40	0.35	6.8%	0.22	0.28	3.3%	0.38	0.39	8.0%
Alcoholic beverages	0.13	0.22	2.3%	0.11	0.21	2.3%	0.18	0.28	2.7%	0.12	0.21	1.9%	0.10	0.18	1.7%	0.15	0.23	2.2%	0.10	0.19	2.0%
Sweetened beverages	0.17	0.27	3.0%	0.17	0.28	3.5%	0.21	0.32	3.1%	0.17	0.25	2.7%	0.15	0.23	2.6%	0.13	0.22	2.0%	0.14	0.22	3.0%
Drinking water	0.12	0.17	2.1%	0.13	0.18	2.7%	0.13	0.18	2.0%	0.11	0.17	1.8%	0.08	0.14	1.3%	0.12	0.15	1.7%	0.12	0.18	2.4%

GHGE greenhouse gas emissions, *SD* standard deviation, *WG* wholegrain.

[1] Percentage of total absolute GHGE.

[2] Meat does not include all consumed meat products, as a small part of meat products is consumed in the form of composite dishes.

[3] Other protein sources include amphibians, reptiles, snails and insects.

* Pairwise t-test comparison, with Bonferroni correction, for impact that was significantly different from all other dietary protein patterns.

Supplemental Table 10 Diet-related land use of the total study population and the six dietary protein patterns, obtained from the EFSA Comprehensive European Food Consumption Database [1].

	**Total**			**Common**			**Fast-food**			**Milk-rich**			**Health-conscious**			**Traditional**			**Plant-forward**		
	Mean	SD	%[1]	Mean	SD	%[1]	Mean	SD	%[1]	Mean	SD	%[1]	Mean	SD	%[1]	Mean	SD	%[1]	Mean	SD	%[1]
**Total LU**																					
Absolute (m^2*year/day)	7.30	3.59		5.86	2.69		9.11	3.39		7.97	4.00		7.46	3.26		8.62	4.30		5.66	2.91	
Standardized (m^2*year/2000 kcal)	7.47	2.94		7.26	2.85		7.67	2.33		7.81	3.41		7.23	2.62		7.99*	3.61		5.57*	2.49	
**Food group LU (m^2*year/day)**																					
Animal-based foods																					
Meat[2]	3.60	3.12	49.3%	2.65	2.28	45.1%	4.75	3.00	52.2%	4.23	3.64	53.1%	3.33	2.80	44.6%	4.77	4.13	55.4%	1.85	2.41	32.8%
Processed meat	0.87	1.10	12.0%	0.53	0.63	9.1%	1.99	1.56	21.9%	0.74	0.87	9.3%	0.76	0.92	10.2%	0.60	0.70	7.0%	0.36	0.72	6.4%
Red meat	2.17	2.84	29.7%	1.62	2.15	27.6%	2.33	2.64	25.6%	2.95	3.47	37.0%	2.00	2.55	26.8%	3.28	3.94	38.1%	1.00	1.88	17.6%
Ruminant meat	1.72	2.73	23.6%	1.28	2.07	21.8%	1.36	2.30	14.9%	2.56	3.40	32.1%	1.68	2.48	22.5%	3.03	3.91	35.2%	0.84	1.78	14.8%
Non-ruminant meat	0.45	0.89	6.2%	0.34	0.66	5.8%	0.97	1.38	10.6%	0.39	0.75	4.9%	0.32	0.67	4.3%	0.25	0.61	2.9%	0.16	0.52	2.8%
White meat	0.48	0.80	6.6%	0.46	0.75	7.9%	0.36	0.70	3.9%	0.51	0.78	6.4%	0.55	0.83	7.3%	0.65	1.00	7.5%	0.48	0.98	8.5%
Offal meat	0.07	0.43	0.9%	0.03	0.22	0.5%	0.07	0.39	0.8%	0.03	0.28	0.4%	0.02	0.19	0.3%	0.24	0.89	2.8%	0.01	0.10	0.2%
Fish and seafood	0.04	0.13	0.6%	0.04	0.11	0.7%	0.01	0.06	0.1%	0.03	0.09	0.4%	0.05	0.13	0.7%	0.08	0.22	0.9%	0.06	0.16	1.0%
Fish	0.03	0.10	0.4%	0.04	0.11	0.6%	0.01	0.06	0.1%	0.02	0.08	0.3%	0.05	0.12	0.6%	0.04	0.12	0.4%	0.04	0.12	0.7%
Seafood	0.01	0.08	0.1%	0.01	0.04	0.1%	0.00	0.02	0.0%	0.01	0.05	0.1%	0.00	0.03	0.1%	0.04	0.18	0.5%	0.02	0.10	0.3%
Other protein sources[3]	0.00	0.00	0.0%	0.00	0.00	0.0%	0.00	0.00	0.0%	0.00	0.00	0.0%	0.00	0.00	0.0%	0.00	0.00	0.0%	0.00	0.00	0.0%
Eggs	0.12	0.20	1.6%	0.07	0.12	1.2%	0.17	0.25	1.8%	0.08	0.13	0.9%	0.19	0.27	2.5%	0.16	0.21	1.8%	0.09	0.15	1.6%
Dairy products	0.85	0.63	11.7%	0.63	0.44	10.8%	0.84	0.62	9.2%	1.09	0.60	13.6%	1.12	0.71	15.0%	1.14	0.77	13.3%	0.72	0.61	12.6%
Milk	0.20	0.25	2.7%	0.13	0.16	2.3%	0.14	0.19	1.5%	0.57	0.38	7.2%	0.25	0.27	3.4%	0.16	0.18	1.8%	0.10	0.18	1.8%
Yoghurt	0.07	0.12	1.0%	0.08	0.12	1.4%	0.05	0.10	0.5%	0.05	0.09	0.6%	0.14	0.19	1.9%	0.05	0.09	0.6%	0.07	0.13	1.3%
Cream and dessert	0.15	0.28	2.0%	0.12	0.22	2.0%	0.14	0.27	1.6%	0.15	0.26	1.9%	0.21	0.35	2.8%	0.18	0.34	2.1%	0.10	0.21	1.8%
Cheese	0.44	0.48	6.0%	0.30	0.32	5.2%	0.51	0.50	5.6%	0.31	0.34	3.9%	0.52	0.51	7.0%	0.76	0.66	8.8%	0.44	0.49	7.8%
Animal fats	0.21	0.37	2.9%	0.18	0.31	3.1%	0.35	0.53	3.9%	0.16	0.31	2.1%	0.15	0.31	2.1%	0.20	0.35	2.4%	0.14	0.30	2.5%
Plant-based foods																					
Grains	1.04	0.79	14.3%	0.91	0.76	15.6%	1.40	0.93	15.4%	0.92	0.67	11.5%	1.09	0.74	14.6%	1.00	0.63	11.6%	0.98	0.78	17.3%
Refined grains	0.95	0.80	13.0%	0.86	0.77	14.7%	1.37	0.93	15.1%	0.81	0.67	10.1%	0.69	0.70	9.2%	0.97	0.63	11.3%	0.83	0.78	14.7%
Breakfast cereals	0.00	0.01	0.0%	0.00	0.01	0.0%	0.00	0.01	0.0%	0.02	0.03	0.3%	0.00	0.01	0.0%	0.00	0.01	0.0%	0.01	0.02	0.1%
Fine bakery wares	0.52	0.74	7.1%	0.53	0.74	9.0%	0.65	0.89	7.2%	0.45	0.63	5.6%	0.47	0.66	6.3%	0.40	0.57	4.6%	0.49	0.72	8.7%
Bread	0.37	0.29	5.1%	0.30	0.20	5.1%	0.67	0.33	7.4%	0.28	0.22	3.5%	0.18	0.18	2.3%	0.41	0.27	4.8%	0.28	0.26	5.0%
Cereals, pasta, rice	0.06	0.08	0.8%	0.04	0.05	0.6%	0.05	0.07	0.5%	0.06	0.07	0.7%	0.04	0.05	0.5%	0.16	0.13	1.8%	0.05	0.07	0.8%
Whole grains	0.09	0.18	1.3%	0.05	0.09	0.9%	0.03	0.09	0.3%	0.11	0.14	1.4%	0.40	0.28	5.4%	0.03	0.07	0.3%	0.15	0.21	2.6%
WG breakfast cereals	0.02	0.08	0.3%	0.01	0.02	0.1%	0.00	0.02	0.0%	0.02	0.05	0.2%	0.10	0.19	1.3%	0.01	0.02	0.1%	0.05	0.14	0.9%
WG bread	0.07	0.15	1.0%	0.04	0.09	0.7%	0.03	0.08	0.3%	0.09	0.13	1.1%	0.29	0.25	3.9%	0.02	0.06	0.2%	0.08	0.13	1.4%
WG cereals, pasta, rice	0.00	0.02	0.0%	0.00	0.01	0.0%	0.00	0.01	0.0%	0.00	0.01	0.0%	0.01	0.03	0.1%	0.00	0.01	0.0%	0.02	0.06	0.4%
Starchy roots and tubers	0.04	0.04	0.5%	0.03	0.04	0.5%	0.04	0.04	0.4%	0.04	0.04	0.6%	0.04	0.04	0.5%	0.03	0.04	0.3%	0.03	0.04	0.5%
Vegetables	0.17	0.35	2.3%	0.18	0.37	3.1%	0.16	0.35	1.8%	0.15	0.30	1.8%	0.16	0.34	2.2%	0.13	0.28	1.5%	0.20	0.37	3.6%
Fruit	0.08	0.09	1.0%	0.08	0.09	1.3%	0.06	0.08	0.6%	0.07	0.08	0.9%	0.09	0.09	1.2%	0.08	0.08	0.9%	0.11	0.11	1.9%

Legumes	0.02	0.10	0.2%	0.01	0.11	0.2%	0.02	0.05	0.2%	0.02	0.09	0.3%	0.02	0.11	0.3%	0.02	0.12	0.2%	0.04	0.12	0.7%
Nuts and seeds	0.04	0.15	0.6%	0.03	0.11	0.5%	0.04	0.14	0.5%	0.03	0.09	0.4%	0.10	0.24	1.3%	0.03	0.12	0.3%	0.19	0.41	3.4%
Vegetable oils and fats	0.24	0.23	3.3%	0.18	0.18	3.1%	0.27	0.26	2.9%	0.22	0.20	2.7%	0.27	0.24	3.6%	0.35	0.25	4.1%	0.23	0.24	4.0%
Meat and dairy imitates	0.01	0.05	0.1%	0.00	0.02	0.0%	0.00	0.01	0.0%	0.00	0.02	0.0%	0.01	0.02	0.1%	0.00	0.01	0.0%	0.30	0.21	5.4%
Meat imitates	0.00	0.03	0.0%	0.00	0.01	0.0%	0.00	0.00	0.0%	0.00	0.01	0.0%	0.00	0.02	0.0%	0.00	0.01	0.0%	0.14	0.21	2.5%
Dairy imitates	0.00	0.03	0.1%	0.00	0.01	0.0%	0.00	0.01	0.0%	0.00	0.01	0.0%	0.00	0.02	0.0%	0.00	0.01	0.0%	0.16	0.18	2.8%
Mixed foods																					
Sugar and confectionary	0.08	0.14	1.2%	0.08	0.14	1.3%	0.09	0.15	1.0%	0.10	0.15	1.2%	0.09	0.14	1.2%	0.07	0.13	0.8%	0.08	0.14	1.5%
Composite dishes	0.28	0.85	3.8%	0.31	0.87	5.3%	0.30	0.85	3.2%	0.36	1.04	4.5%	0.24	0.81	3.2%	0.14	0.59	1.7%	0.23	0.74	4.0%
Miscellaneous	0.12	0.27	1.6%	0.11	0.24	1.9%	0.12	0.22	1.3%	0.13	0.28	1.7%	0.17	0.40	2.2%	0.10	0.28	1.1%	0.12	0.26	2.1%
Beverages																					
Hot beverages	0.16	0.20	2.2%	0.17	0.20	2.8%	0.18	0.22	1.9%	0.16	0.20	2.0%	0.18	0.19	2.5%	0.12	0.15	1.3%	0.13	0.16	2.2%
Alcoholic beverages	0.10	0.20	1.4%	0.08	0.17	1.4%	0.17	0.29	1.8%	0.09	0.19	1.1%	0.08	0.16	1.0%	0.10	0.18	1.2%	0.07	0.14	1.1%
Sweetened beverages	0.12	0.18	1.6%	0.11	0.18	1.9%	0.14	0.21	1.6%	0.12	0.17	1.5%	0.11	0.16	1.4%	0.10	0.16	1.1%	0.10	0.15	1.7%
Drinking water	0.00	0.00	0.0%	0.00	0.00	0.0%	0.00	0.00	0.0%	0.00	0.00	0.0%	0.00	0.00	0.0%	0.00	0.00	0.0%	0.00	0.00	0.0%

LU land use, *SD* standard deviation, *WG* wholegrain.

[1] Percentage of total absolute LU.

[2] Meat does not include all consumed meat products, as a small part of meat products is consumed in the form of composite dishes.

[3] Other protein sources include amphibians, reptiles, snails and insects.

* Pairwise t-test comparison, with Bonferroni correction, for impact that was significantly different from all other dietary protein patterns.

References

[1] European Food Safety Authority (EFSA), Food consumption data. https://www.efsa.europa.eu/en/data-report/food-consumption-data, 2022 (accessed 2 January 2023).

[2] European Food Safety Authority (EFSA), 2017. Dietary Reference Values for nutrients Summary report. EFSA Supporting Publications. 14, e15121E. https://doi.org/10.2903/SP.EFSA.2017.E15121.

[3] R. Blomhoff, R. Andersen, E.K. Arnesen, J.J. Christensen, H. Eneroth, M. Erkkola, I. Gudanaviciene, T.I. Halldorsson, A. Høyer-Lund, E.W. Lemming, H.M. Meltzer, T. Pitsi, U. Schwab, I. Siksna, I. Thorsdottir, E. Trolle, Nordic Nutrition Recommendations 2023, Copenhagen, 2023. https://www.norden.org/en/publication/nordic-nutrition-recommendations-2023.

[4] World Health Organization (WHO), Guideline: sugars intake for adults and children, Geneva, 2015. https://www.who.int/publications/i/item/9789241549028.

[5] Food and Agriculture Organization (FAO), Fats and fatty acids in human nutrition. Report of an expert consultation., FAO Food Nutr Pap. 91 (2010) 1–166. https://europepmc.org/article/med/21812367.

[6] World Health Organization (WHO), Guideline: sodium intake for adults and children, Geneva, 2012. https://www.who.int/publications/i/item/9789241504836.

[7] World Health Organization (WHO), A healthy lifestyle - WHO recommendations. https://www.who.int/europe/news-room/fact-sheets/item/a-healthy-lifestyle---who-recommendations, 2010 (accessed 10 August 2023).

[8] European Union, EU Vocabularies. https://op.europa.eu/en/web/eu-vocabularies/concept-scheme/-/resource?uri=http://eurovoc.europa.eu/100277, n.d. (accessed 13 December 2023).
